# Thickness and defect dependent electronic, optical and thermoelectric features of $\hbox {WTe}_2$

**DOI:** 10.1038/s41598-022-16899-5

**Published:** 2022-07-26

**Authors:** Ilkay Ozdemir, Alexander W. Holleitner, Christoph Kastl, Olcay Üzengi Aktürk

**Affiliations:** 1 Physics Department, Adnan Menderes University, 09100 Aydin, Turkey; 2 Walter Schottky Institut and Physics Department, Technical University of Munich, Am Coulombwall 4a, 85748 Garching, Germany; 3 Munich Center of Quantum Science and Technology (MCQST), Schellingstr. 4, 80799 Munich, Germany; 4 Electrical Electronics Engineering Department, Adnan Menderes University, 09100 Aydin, Turkey

**Keywords:** Nanoscience and technology, Nanoscale materials, Two-dimensional materials

## Abstract

Transition metal dichalcogenides (TMDs) receive significant attention due to their outstanding electronic and optical properties. In this study, we investigate the electronic, optical, and thermoelectric properties of single and few layer $\hbox {WTe}_2$ in detail utilizing first-principles methods based on the density functional theory (DFT). Within the scope of both PBE and HSE06 including spin orbit coupling (SOC), the simulations predict the electronic band gap values to decrease as the number of layers increases. Moreover, spin-polarized DFT calculations combined with the semi-classical Boltzmann transport theory are applied to estimate the anisotropic thermoelectric power factor (Seebeck coefficient, *S*) for $\hbox {WTe}_2$ in both the monolayer and multilayer limit, and *S* is obtained below the optimal value for practical applications. The optical absorbance of $\hbox {WTe}_2$ monolayer is obtained to be slightly less than the values reported in literature for 2H TMD monolayers of $\hbox {MoS}_2$, $\hbox {MoSe}_2$, and $\hbox {WS}_2$. Furthermore, we simulate the impact of defects, such as vacancy, antisite and substitution defects, on the electronic, optical and thermoelectric properties of monolayer $\hbox {WTe}_2$. Particularly, the Te-$\hbox {O}_2$ substitution defect in parallel orientation yields negative formation energy, indicating that the relevant defect may form spontaneously under relevant experimental conditions. We reveal that the electronic band structure of $\hbox {WTe}_2$ monolayer is significantly influenced by the presence of the considered defects. According to the calculated band gap values, a lowering of the conduction band minimum gives rise to metallic characteristics to the structure for the single Te(1) vacancy, a diagonal Te line defect, and the Te(1)-$\hbox {O}_2$ substitution, while the other investigated defects cause an opening of a small positive band gap at the Fermi level. Consequently, the real ($\varepsilon _1(\omega )$) and imaginary ($\varepsilon _2(\omega )$) parts of the dielectric constant at low frequencies are very sensitive to the applied defects, whereas we find that the absorbance (*A*) at optical frequencies is less significantly affected. We also predict that certain point defects can enhance the otherwise moderate value of *S* in pristine $\hbox {WTe}_2$ to values relevant for thermoelectric applications. The described $\hbox {WTe}_2$ monolayers, as functionalized with the considered defects, offer the possibility to be applied in optical, electronic, and thermoelectric devices.

## Introduction

Two-dimensional (2D) layered materials have become a versatile experimental and theoretical platform to reveal how physical phenomena change and how new physical properties emerge when the dimension of a crystal structure is reduced from bulk to a single atomic layer. Within this scope, monolayer transition metal dichalcogenides (TMDs) with the general chemical formula of $\hbox {MX}_2$, where M is a transition metal atom (Mo, W, etc.) and X is a chalcogen atom (S, Se, or Te), have turned into an attractive research field in condensed matter physics due to their promising electronic, spintronic, and optical properties^1–5^.

Bulk TMDs cover almost all known condensed matter phases, including insulators (e.g. $\hbox {HfS}_2$), semiconductors (e.g. $\hbox {MoS}_2$ and $\hbox {WS}_2$), semimetals (e.g. $\hbox {WTe}_2$ and $\hbox {TiSe}_2$), as well as metals (e.g. $\hbox {NbS}_2$ and $\hbox {VSe}_2$)^1^. The bulk TMDs further comprise materials with superconducting and topological electronic properties^6–8^. In their bulk form, TMDs exhibit layered crystal structures reminiscent of graphite with weak van der Waals interlayer interactions between successive $\hbox {MX}_2$ sheets allowing the TMDs to be delaminated and exfoliated down to a single layer^9^. The typical bulk phases of TMDs are 1T, 2H and 3R^1,10,11^. In these phases, layers are stacked in the sequence of *AbC* with an octahedral symmetry, *AbA BaB* with a hexagonal symmetry, and *AbA CaC BcB* with a rhombohedral symmetry, respectively. Whilst the metal atom coordination in the 2H and 3R phases is trigonal prismatic, it is trigonal anti-prismatic (or octahedral) in the 1T phase. The 1T phase is known to be a metastable form, which, in free-standing conditions, tends to undergo a spontaneous lattice distortion through dimerization of transition metal atoms along one of the lattice directions. This dimerization lowers the symmetry of the crystal lattice resulting in anisotropic electronic properties^2^. The atomic structure of the distorted (or dimerized) plane can be regarded as one-dimensional dimers of transition metal atoms sandwiched by two one-dimensional zigzag chains of chalcogen atoms. Bulk crystals further distinguish an overall inversion symmetric (1T′) and an inversion symmetry broken stacking order ($\hbox {T}_d$). The most prominent example is $\hbox {WTe}_2$ and its cousin $\hbox {MoTe}_2$. While $\hbox {WTe}_2$ crystallizes in the $\hbox {T}_d$ phase already at room temperature^12^, $\hbox {MoTe}_2$ undergoes a phase transition from the 1T′ to $\hbox {T}_d$ phase at about ${250}\hbox {K}$^13^. In their bulk form, both materials are semimetals with intriguing physical properties dictated by the reduced crystal symmetry and almost perfect charge carrier compensation. For example, it has been reported experimentally that bulk $\hbox {WTe}_2$ possesses a large and non-saturating positive magnetoresistance^14,15^, pronounced spin-orbit texture^16^, pressure-induced superconductivity^17,18^, unconventional Nernst response^19^ and low-energy optical absorption^20^. Later on, through first principles calculations, Weyl states were predicted in bulk $\hbox {WTe}_2$, $\hbox {MoTe}_2$ as well as their alloy ($\hbox {Mo}_x\hbox {W}_{1-x}\hbox {Te}_2$), indicating that they are candidates to realize a type-II topological Weyl semimetal phase^21–23^. The existence of type-II Weyl points in $\hbox {WTe}_2$ means that many of its physical properties are very different to those of standard Weyl semimetals with point-like Fermi surfaces^21^. The Weyl phase is further connected to the existence of strong Berry curvatures in the Brillouin zone of bulk $\hbox {WTe}_2$, which can give rise to nonlinear and optically induced Hall effects along specific crystal directions with reduced symmetry^24–27^. In the monolayer limit $\hbox {WTe}_2$ has T′ structure^28^, and it has been predicted by density functional theory (DFT) to be in a quantum spin Hall phase (QSH) through opening of an inverted band gap^2^. Zheng et al. performed calculations based on hybrid functional methods beyond standard DFT and obtained a positive QSH band gap in monolayer 1T′ $\hbox {WTe}_2$^29^. Moreover, they predicted an increase of the band gap with decreasing layer number, and they reported first experimental evidence that suggested an opening of a bulk gap in the few layer limit. Since then, the QSH phase of monolayer $\hbox {WTe}_2$ has been firmly established by the experimental observation of edge states, measurements of quantized edge conductance and measurements of the edge states’ spin-polarization^28,30–34^. Overall, these unconventional quantum properties render $\hbox {WTe}_2$ appealing for potential applications in nanotechnology.

In addition to dimensionality and symmetry, the electronic and optical properties of 2D materials are particularly susceptible to lattice defects. In fact, it is inevitable that atomic-scale point defects occur within the fabrication process of 2D materials using, for example, mechanical exfoliation or chemical vapor deposition (CVD)^35^. While defects are considered usually detrimental, they also provide an opportunity to modify material properties and to create new functionality, an approach which has been coined defect engineering^36,37^. Lithographic methods, such as focused ion beam microscopy, have been shown to enable targeted modification of 2D materials down to the limit of single point defects^38^. For the case of monolayer $\hbox {WTe}_2$, the effect of selected point defects on the electronic structure and topological properties has been studied recently^39^. It was found that while vacancies strongly influence the band structure, adatoms do not change the electronic structure in the vicinity of the Fermi level and thus the topological properties^39^. Yet so far, the layer dependent properties and the effects of point defects on the monolayer of $\hbox {WTe}_2$ remain to be elucidated in detail, in light of their possible application in nanoscale devices. Here, using first principles methods, we elucidate the anisotropic electronic, optical, and thermoelectric properties of monolayer and multilayer 1T′ $\hbox {WTe}_2$ in equilibrium and reveal the effects of various point defects on monolayer 1T′ $\hbox {WTe}_2$.

## Results

We organized the main part of this study into two subsections: (*i*) Revealing the structural, electronic, optical and thermoelectric properties of monolayer and multilayer $\hbox {WTe}_2$ in equilibrium, and (*ii*) evaluation of the aforementioned properties under various point defects for monolayer $\hbox {WTe}_2$.

### Monolayer and multilayer 1T′ $\hbox {WTe}_2$ in equilibrium

**Figure 1:**
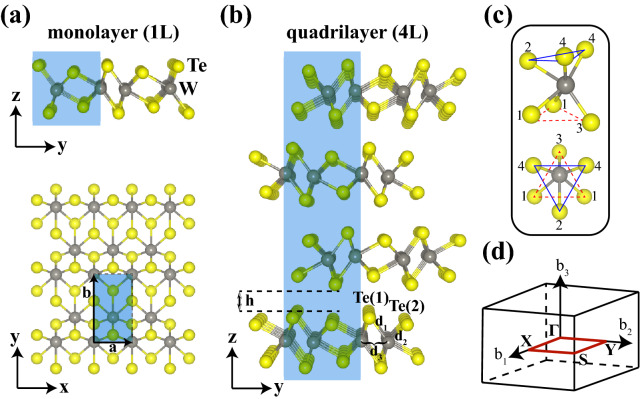
Optimized crystal structures with (**a**) side view (*yz*-plane) and top view (*xy*-plane) of monolayer (1L) and (**b**) side view of quadrilayer (4L) 1T′ $\hbox {WTe}_2$. The rectangular unit cells are shown by the blue-shaded areas omitting the vacuum distance in the *z*-direction. (**c**) Distorted octahedral geometry formed by one W and six Te atoms with two different views, where pairs of rotated triangles are indicated with blue-solid and red-dashed lines. (**d**) 3D orthorhombic Brillouin zone (BZ), in which the 2D BZ is indicated by the red-rectangle with corresponding high symmetry points, i.e. $\Gamma$, X, S, Y.

As is known from earlier experimental and theoretical studies, three dimensional (3D) $\hbox {WTe}_2$ crystallizes in the distorted 1T structure ($\hbox {T}_d$)^14,15,21,40^. It belongs to the $C_{2\nu }(mm2)$ point group in the $Pmn2_1$ space group^40^. As the dimension is reduced to 2D, i.e. from bulk through few-layer to monolayer, both structural and electronic properties of $\hbox {WTe}_2$ change and the structure emerges in 1T′ phase. In accordance with literature^41^, we obtained that bulk $\hbox {WTe}_2$ is of $C_{2\nu }$ symmetry, while few-layer has $C_{s}$ and monolayer has $C_{2h}$ symmetry. Experimentally, single and few-layer samples can be obtained through mechanical exfoliation from bulk crystals^42^. Theoretically, we obtained geometric structures of monolayer and few-layer $\hbox {WTe}_2$ by removing the redundant layers from the bulk structure and employing a minimum vacuum distance of ${15}{\text{\AA} }$ along the *z*-lattice direction. In Fig. 1a,b, we present the optimized atomic configurations of monolayer (1L) and quadrilayer (4L) 1T′ $\hbox {WTe}_2$ structures.

The structure of a single $\hbox {WTe}_2$ layer consists of three, covalently bonded, atomic planes which are stacked in the order of Te-W-Te along the *z*-axis. Each W atom forms a triangular pyramid with the three nearest Te atoms from both layers above and below. On opposing sides, these pyramids are rotated ${180}^{\circ }$ (about the *z*-axis) relative to each other^41^ (Fig. 1c). We calculated the distortion of W atoms, predominantly caused by the convergence of metal atoms to each other under the influence of strong intermetallic bonding, to be ${0.87}{\text{\AA} }$ along the *y*-direction and ${0.21}{\text{\AA} }$ along the *z*-direction, in good agreement with both experimental and theoretical literature reports^43–45^. Within the distorted $\hbox {WTe}_2$ structure, Te atoms are not located in a coplanar plane. Instead, they form a zigzag chain along the *y*-direction. The calculated buckling distance along the *z*-direction is ${0.6}{\text{\AA} }$, which is consistent with literature findings^45^.

In multilayer 1T′ $\hbox {WTe}_2$, the arrangement of adjacent, stacked layers with respect to each other is reminiscent of the lock and key model, i.e. ripples of one layer correspond to grooves of the other one and vice versa. Accordingly, successive layers stand rotated ${180}^{\circ }$ relative to each other around the *z*-axis. The interaction between adjacent $\hbox {WTe}_2$ layers is of weak van der Waals type, and the interlayer distance (*h*) is also called the van der Waals distance. We summarized the structural parameters, including lattice parameters, bond lengths between adjacent W-Te and W-W atoms, van der Waals distances between layers, cohesive energies per atom calculated for monolayer and multilayer structures of 1T′ $\hbox {WTe}_2$ in Table 1.

**Table 1:** Structural parameters calculated within PBE+SOC for 1L, 2L, 3L, 4L, 5L and 6L 1T′ $\hbox {WTe}_2$: Lattice constants in the (*xy*)-plane, $\mid \vec {a} \mid$ = a ($\parallel x$) and $\mid \vec {b} \mid$ = b ($\parallel y$); interlayer (van der Waals) distance between successive $\hbox {WTe}_2$ layers, *h*; bond lengths between neighboring W and Te atoms, $d_1$ (W-Te(1)), $d_2$ (W-Te(2)), and between W atoms $d_3$ (W-W); cohesive energy per atom, $E_{\text {coh}}$ (eV/atom).

Structure	a (Å)	b (Å)	h (Å)	$\hbox {d}_1$ (Å)	$\hbox {d}_2$ (Å)	$\hbox {d}_3$ (Å)	$\hbox {E}_{\text {coh}}$
1L-$\hbox {WTe}_2$	3.508	6.231	–	2.721	2.816	2.857	4.778
2L-$\hbox {WTe}_2$	3.529	6.232	2.745	2.721	2.823	2.861	4.847
3L-$\hbox {WTe}_2$	3.534	6.230	2.732	2.720	2.824	2.862	4.874
4L-$\hbox {WTe}_2$	3.534	6.230	2.730	2.721	2.824	2.861	4.884
5L-$\hbox {WTe}_2$	3.535	6.230	2.733	2.721	2.824	2.861	4.894
6L-$\hbox {WTe}_2$	3.536	6.231	2.732	2.718	2.821	2.862	4.899

**Figure 2:**
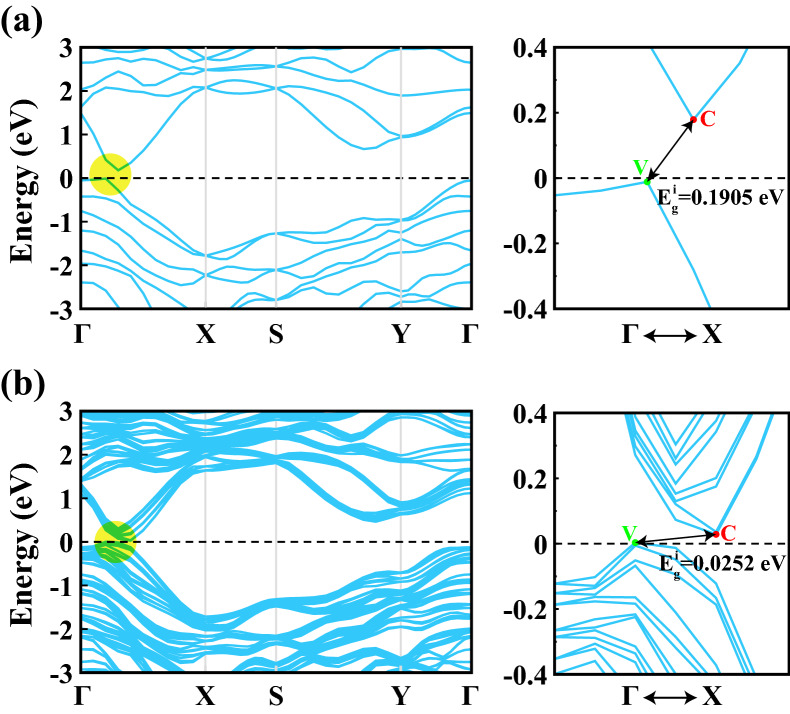
Electronic energy band structures, $E_n({{\textbf {k}}})$, calculated with the HSE06 functional including SOC along major symmetry directions of the 2D Brillouin zone for (**a**) 1L and (**b**) 4L 1T′ $\hbox {WTe}_2$. The Fermi level (black-dashed line) is set to zero energy. The arrows highlight the indirect band gaps ($\hbox {E}_{\text {g}}^{\text {i}}$) occurring along the $\Gamma -X$ direction. The conduction band minimum and valence band maximum are indicated by C and V, respectively.

Figure 2 shows the electronic energy band structures of 1L and 4L 1T′ $\hbox {WTe}_2$ calculated using the HSE06 functional^46^ including SOC along major symmetry directions of the 2D Brillouin zone (BZ). We also calculated the electronic energy band structures and corresponding density of states (DOS) of monolayer and multilayer 1T′ $\hbox {WTe}_2$ by PBE functional without and with SOC, and band structures of 2L and 3L $\hbox {WTe}_2$ within HSE06 functional, which are presented in the Supporting Information. From 1L to 4L, $\hbox {WTe}_2$ exhibits a qualitatively similar electronic structure and is a narrow gap semimetal, as expected from literature findings^29,47^. In all cases, electron states and hole states form small pockets around the valence band maximum (VBM) and conduction band minimum (CBM), respectively. The centers of the electron and hole pockets differ slightly along $\Gamma$-*X* direction, which is the direction along W-W dimerization in real space, yielding the narrow indirect band gaps, which are also highlighted in Fig. 2. The band gap values calculated by PBE and HSE06 functionals without and with SOC parameter are tabulated in Table 2. We define the band gap as the energy difference between the conduction band minimum and valence band maximum, $E_{\text {g}} = E_{\text {CBM}} - E_{\text {VBM}}$. Therefore, positive values correspond to an finite energy gap between the electron and hole pockets, and negative values correspond to an energetic overlap of electron and hole pockets. Contrary to PBE, which tends to underestimate the band gap, the HSE06 functional including SOC, which is known to estimate the band gap more accurately, yields positive band gap values for monolayer and multilayer $\hbox {WTe}_2$^2,29,32^. The calculated band gap values decrease with increasing number of layers both for HSE06 and PBE functionals. At the PBE level, SOC shows the effect of reducing the band gap from 1L to 3L (making it more negative), while increasing (closer to zero) it at 4L and beyond. Within the HSE06 calculations, SOC causes a band gap opening and it gives always rise to an increase of the band gap. We found slightly different band gap values from the ones obtained by Zheng et al.^29^. The differences mainly arise from number of **k**-points, different functionals, and parameters used in the calculations.

**Table 2:** Electronic band gap values (in eV) calculated within PBE and HSE06 functionals without and with SOC for 1L, 2L, 3L and 4L 1T′ $\hbox {WTe}_2$. The band gap is calculated as the energy difference between the conduction band minimum and valence band maximum, $E_{\text {CBM}} - E_{\text {VBM}}$.

Structure/Method	PBE	PBE+SOC	HSE	HSE+SOC
1L-$\hbox {WTe}_2$	0	− 0.0052	0.1191	0.1905
2L-$\hbox {WTe}_2$	− 0.0221	− 0.0397	0.0463	0.1172
3L-$\hbox {WTe}_2$	− 0.0761	− 0.0773	− 0.0297	0.0645
4L-$\hbox {WTe}_2$	− 0.1123	− 0.0903	− 0.0712	0.0252

**Figure 3:**
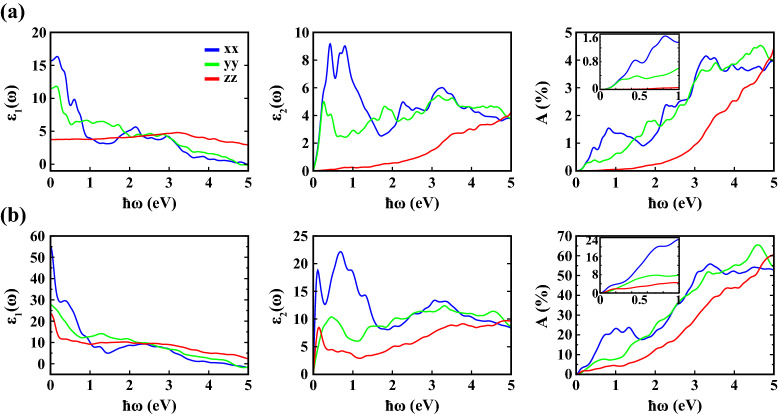
Real ($\varepsilon _1(\omega )$) and imaginary ($\varepsilon _2(\omega )$) parts of the frequency dependent dielectric constant, and absorbance (*A*) calculated within PBE+SOC for (**a**) 1L and (**b**) 4L 1T′ $\hbox {WTe}_2$ as a function of photon energy ($\hbar \omega$) at 0 K along different crystallographic directions (*xx*, *yy*, *zz*).

In Fig. 3, we present the real ($\varepsilon _1(\omega )$) and imaginary ($\varepsilon _2(\omega )$) parts of the complex dielectric constant as well as absorbance (*A*) as a function of photon energy ($\hbar \omega$) for 1L and 4L 1T′ $\hbox {WTe}_2$ within PBE+SOC. The corresponding calculations for 2L and 3L are presented in the Supporting Information. We calculated the optical parameters at 0 K, within the photon energy range of 0–5 eV, along in-plane ($\bf E\parallel x$ and $\bf E\parallel y$) and out-of-plane ($\bf E\parallel z$) directions. The anisotropic crystal structure of 1T′ $\hbox {WTe}_2$, where the W-W dimerization breaks the symmetry of the structure, is also reflected in anisotropic in-plane optical properties along the corresponding lattice directions.

The qualitative characteristics of the optical response above 1 eV are largely unaffected by the number of layers, but overall the dielectric values increase. We calculated the static dielectric constants along the *xx* direction (i.e. $\varepsilon ^{xx}_1(0)$) as 15.7, 28.4, 44.5 and 55.1 for 1L, 2L, 3L and 4L $\hbox {WTe}_2$, respectively. The values obtained are inversely proportional to the electronic energy band gap values, obtained between $\Gamma$-*X* points for $\hbox {WTe}_2$, presented in Table 2. This result is similar to Penn’s model for semiconductors^48^.

As for the calculated $\varepsilon _2(\omega )$ along the crystallographic *x* axis, which arises from interband transitions, we observed two main peaks within the spectrum, which are located between 0–1 eV for all structures (i.e. 1L, 2L, 3L, 4L $\hbox {WTe}_2$) with different intensities. Besides, while the location of the second peak remains almost constant as the number of layers increases, the first peak moves to lower energies associated with the decreasing electronic energy band gap values provided in Table 2. The intensities and locations of the first and second peaks of $\varepsilon _2^{xx}(\omega )$ are calculated as 9.2 at 0.431 eV and 9.0 at 0.804 eV for 1L $\hbox {WTe}_2$, 15.2 at 0.377 eV and 13.8 at 0.754 eV for 2L $\hbox {WTe}_2$, 18.7 at 0.392 eV and 17.7 at 0.769 eV for 3L $\hbox {WTe}_2$, 18.8 at 0.113 eV and 22.2 at 0.696 eV for 4L $\hbox {WTe}_2$, respectively.

With the real ($\varepsilon _1(\omega )$) and imaginary ($\varepsilon _2(\omega )$) parts of the complex dielectric constant, we derive the frequency-dependent optical absorbance of 1T′ $\hbox {WTe}_2$, which is a typical experimental parameter relevant to the identification of thin layer samples. We used Eq. (5), which is an adequate approximation for ultra thin materials ($\Delta z \rightarrow 0$) on transparent substrates, which has been validated by Bernardi et al.^49^ for monolayer $\hbox {MoS}_2$ and by Ersan et al.^50^ for monolayer graphene. This equation can be considered as the Taylor expansion of the expression $A(\omega )=1-e^{-\alpha \Delta z}$. Here, $\alpha$ is the absorption coefficient^51^ and $\Delta z$ is the length of the simulation cell normal to the surface, taken as the thickness of the structure in this study. Figure 3 presents the absorbance spectra of 1L and 4L 1T′ $\hbox {WTe}_2$ as a function of photon energy ($\hbar \omega$) within the range of 0–5 eV. Within PBE, our first principles calculations show that monolayer 1T′ $\hbox {WTe}_2$ possesses an optical absorbance of approximately 1% to 4% in the visible range along *xx* and *yy* directions. For comparison, monolayer, semiconducting TMDs, e.g. $\hbox {MoS}_2$, $\hbox {MoSe}_2$, $\hbox {WS}_2$ exhibit an absorbance of about 5-10% in the visible range, as excitonic resonances greatly enhance the light matter interaction^49^. The absorbance of $\hbox {WTe}_2$ increases with layer number not only due to the increase in structural thickness, but also due to the decreasing band gap (cf. Table 2) and the concomitant increase in dielectric constant.

**Figure 4:**
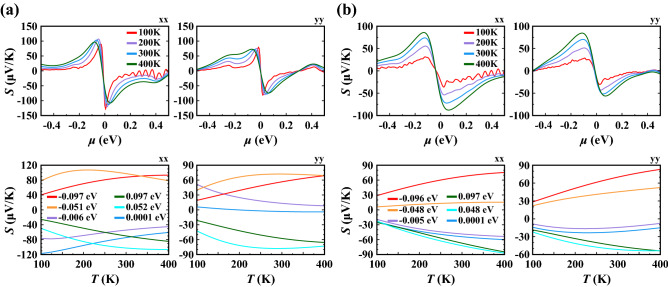
Seebeck coefficient (*S*) calculated as a function of chemical potential ($\mu$) at various temperatures and as a function of temperature (*T*) at various chemical potentials for (**a**) 1L and (**b**) 4L 1T′ $\hbox {WTe}_2$ along both *xx* and *yy* lattice directions.

**Figure 5:**
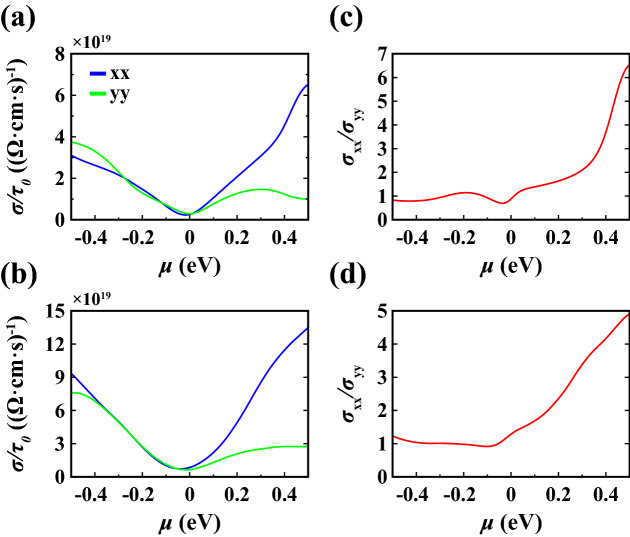
Electrical conductivity with respect to constant relaxation time ($\sigma /\tau _0$) calculated as a function of chemical potential ($\mu$) at 300 K for (**a**) 1L and (**b**) 4L 1T′ $\hbox {WTe}_2$ along *xx* and *yy* lattice directions. Anisotropy factor $\sigma _{xx}/\sigma _{yy}$ of electrical conductivity for (**c**) 1L and (**d**) 4L 1T′ $\hbox {WTe}_2$.

Next, we examined the anisotropic thermoelectric transport properties of 1T′ $\hbox {WTe}_2$ by calculating the Seebeck coefficient (*S*) and electrical conductivity with respect to relaxation time ($\sigma /\tau _0$) as functions of chemical potential ($\mu$) and temperature (*T*) using the BoltzTraP2 code^52^ (Figs. 4, 5). The transport calculations are based on band structures obtained by the PBE functional. Therefore, we restrict our analysis to elevated temperatures, where we can expect significant thermal activation of carriers across the small band gaps predicted by the HSE06 functional. The Seebeck coefficients (*S*) of 1L and 4L 1T′ $\hbox {WTe}_2$ obtained along the *xx* and *yy* directions are presented in Fig. 4 as a function of the chemical potential ($\mu$) for various temperatures (*T*) and as a function of the temperature (100–400 K) for selected chemical potentials (for the data on 2L and 3L $\hbox {WTe}_2$ see Supporting Information). The thermopower shows a characteristic sign change and corresponding maxima near $\mu = 0$ (Fig. 4a,b) due to the reversal of the dominant charge carrier type from holes to electrons, as expected for a semimetal or small gap semiconductor. We note that due to the electron hole asymmetry in the system, the value of $\mu$ where *S* is zero is not equivalent to the charge neutrality point. Contrary to graphene^53^, the change in Seebeck coefficient of $\hbox {WTe}_2$ with temperature for the chemical potentials considered shows non-linear characteristics in the *S*-*T* spectrum, which arises again from the fact that at elevated temperatures carriers are activated across the small/nonexisting gap, such that there is a competition between the transport properties of the non-symmetric electron and hole pockets. We note that phonon drag caused by interband transitions between two linear bands can also give rise to a nonlinear temperature dependence in the thermopower^54^. However, this effect is not considered in our calculations because the electron-phonon coupling is expected to be weak for 1T′ $\hbox {WTe}_2$^55,56^.

The calculated maximum values of *S*, tabulated in the Supporting Information, are in the range of 51–113 $\upmu \hbox {V}\,\hbox {K}^{-1}$. Overall, we find that with increasing number of layers, the maximum value of *S* decreases. In general, thermoelectric materials relevant for applications have a Seebeck coefficient of ${200}\,\upmu \hbox {V}\,\hbox {K}^{-1}$ and above^57^. For thermoelectric applications, the properties of $\hbox {WTe}_2$ may be tuned by external factors, e.g. defect or strain engineering.

Electrical conductivities with respect to constant relaxation time ($\sigma /\tau _0$) calculated for 1L and 4L 1T′ $\hbox {WTe}_2$ as a function of chemical potential ($\mu$) at 300 K are depicted in Fig. 5a,b (see Supporting Information for $\sigma /\tau _0$ of 2L and 3L $\hbox {WTe}_2$). As expected, the electrical conductivity is minimized near $\mu ={0}$ eV. We note that, within the PBE approximation, the conductivity remains finite even at low temperatures due to the absence of a full gap. Notably, the calculations (Fig. 5c,d) show that the electrical conductivity is almost isotropic for negative chemical potential (hole doping) and highly anisotropic for positive chemical potential (electron doping).

### Characteristics of monolayer 1T′ $\hbox {WTe}_2$ under point defects

**Figure 6:**
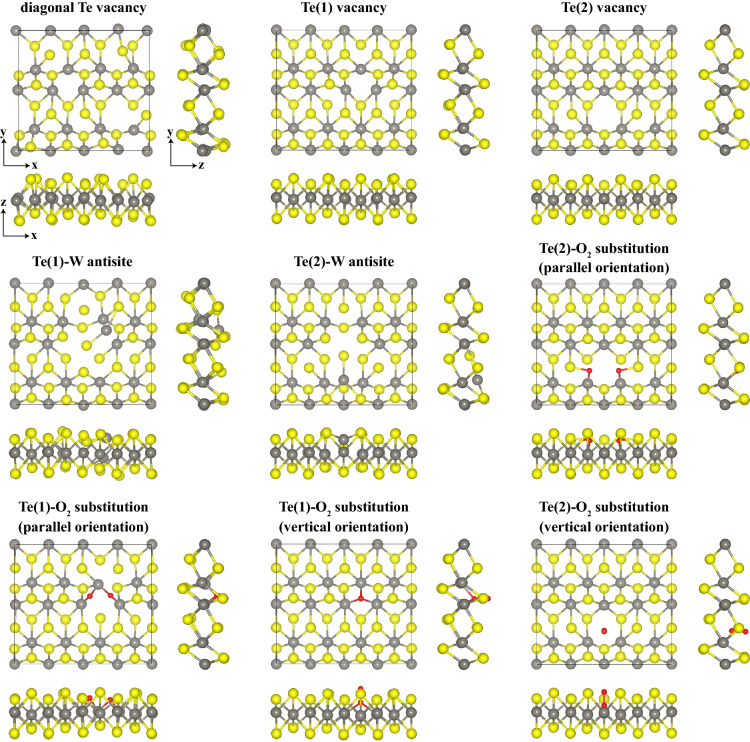
Ball and stick model of optimized atomic structures of defective 1L 1T′ $\hbox {WTe}_2$. W atoms are displayed in grey, Te atoms are displayed in yellow, and O atoms are displayed in red.

Next, we discuss the effects of various point defects on the electronic, optical and thermoelectric characteristics of 1L 1T′ $\hbox {WTe}_2$ by DFT calculations. As it is clear from Fig. 1, 1T′ $\hbox {WTe}_2$ has two non-equivalent Te atoms on its outermost surface, which we label Te(1) and Te(2). We considered two distinct types of vacancies, which are a single Te(1) vacancy and a single Te(2) vacancy. Furthermore, we studied a Te vacancy line defect, where Te atoms are removed diagonally in the unit cell. More complex defect geometries, such as the latter one, effectively further reduce the symmetry of the crystal, and it is envisioned that they can be fabricated experimentally by atom scale fabrications methods, such as focused electron or ion microscopy as well as scanning probe microscopy^38,58,59^. For the antisite defects, labelled A1 and A2, we investigated Te(1)-W and Te(2)-W antisites, where the positions of a Te(1) and a Te(2) atom with the neighboring W atom are exchanged. Finally, we also considered substitutional defects, where an oxygen molecule ($\hbox {O}_2$) replaces a Te atom. For this type of defect, four distinct geometries were considered: a Te(1)-$\hbox {O}_2$ substitution with the oxygen molecule oriented parallel (perpendicular) to the surface and a Te(2)-$\hbox {O}_2$ substitution with the oxygen molecule oriented parallel (perpendicular) to the surface. The investigated point defects are shown in Fig. 6 with their optimized atomic configurations. Initial structures are presented in the Supporting Information.

First, we calculated the cohesive ($E_{\text {coh}}$) and formation ($E_{\text {for}}$) energies for each structure by using Eqs. (1) and (2) (Table 3). The formation energies of A1 and A2 are the same in Te-rich and W-rich environments, since there is neither subtraction nor addition of atoms to the system. Since Te reaches its maximum chemical potential value in Te-rich condition, the calculated formation energies for Te-vacancy-containing defects in Te-rich environment are smaller than those obtained in W-rich environment. While the antisite defects cause expansion in lattice parameters in both *x* and *y* directions, Te vacancies generally cause shrinkage. We note that when the $\hbox {O}_2$ molecule was placed in the location of Te vacancy, both small fluctuating expansions and contractions in the lattice parameters occurred. Besides, it is noteworthy that the formation energy of $\hbox {O}_2$ substitution defect in parallel orientation is negative indicating that this defect may form spontaneously under relevant experimental conditions, i.e. for defective $\hbox {WTe}_2$ exposed to ambient conditions. As can be seen from the relaxed structures, in this configuration, the oxygen molecule undergoes a dissociation. For Te(1) vacancy sites, each oxygen atom binds individually to two neighboring W atoms, whereas for Te(2) vacancy sites, each oxygen atom binds covalently to one W atom and one Te atoms. By contrast, in the vertical configuration, the oxygen molecule is not dissociated, but it rather binds to three neighboring W (Te) atoms by means of a local charge transfer creating an $\hbox {O}_{2-}$ complex (see Supporting Information).

**Table 3:** Cohesive energy ($E_{\text {coh}}$) per atom, formation energy calculated in Te-rich environment ($E_{\text {for}}^{\text {Te}}$) and in W-rich environment ($E_{\text {for}}^{\text {W}}$), electronic energy band gap ($E_{\text {g}}$) values calculated for defective structures of 1L $1T'$ $\hbox {WTe}_2$. Abbreviations used in the first column of the table are as follows: diagTe, diagonal Te vacancy; Te1, Te(1) vacancy; Te2, Te(2) vacancy; A1, Te(1)-W antisite; A2, Te(2)-W antisite; Sub1par, Te(1)-$\hbox {O}_2$ substitution with parallel orientation; Sub1ver, Te(1)-$\hbox {O}_2$ substitution with vertical orientation; Sub2par, Te(2)-$\hbox {O}_2$ substitution with parallel orientation; Sub2ver, Te(2)-$\hbox {O}_2$ substitution with vertical orientation. Note that, M denotes the metallic case, in which some of the electronic bands cross the Fermi energy ($E_{\text {F}}$).

1L 1T′ $\hbox {WTe}_2$				
	$\hbox {E}_{\text {coh}}$ (eV/atom)	$\hbox {E}_{\text {for}}^{\text {Te}}$ (eV)	$\hbox {E}_{\text {for}}^{\text {W}}$ (eV)	$\hbox {E}_{\text {g}}$ (eV)
diagTe	4.789	2.195	2.358	M
Te1	4.774	2.528	2.691	M
Te2	4.777	2.368	2.531	0.028
A1	4.659	5.740	5.740	0.036
A2	4.668	5.291	5.291	0.041
Sub1par	4.795	− 2.026	− 1.863	0.058
Sub1ver	4.753	0.043	0.206	M
Sub2par	4.770	− 0.798	− 0.635	0.006
Sub2ver	4.744	0.471	0.635	0.017

**Figure 7:**
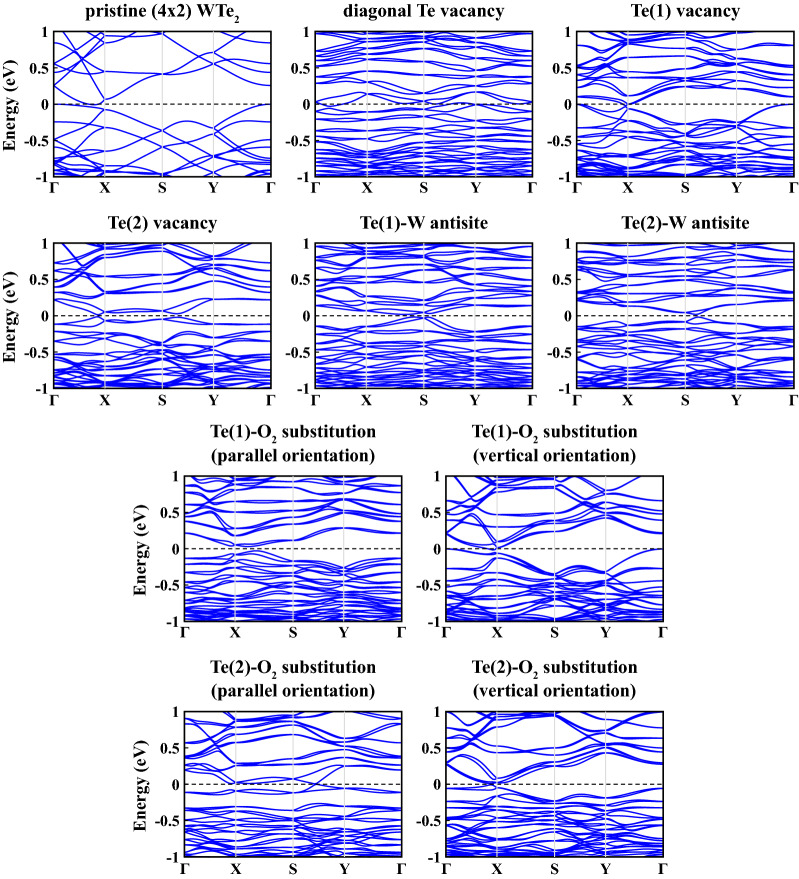
Electronic energy band structures, $E_n({{\textbf {k}}})$, of defective $1T'$ $\hbox {WTe}_2$ monolayer calculated within PBE+SOC along major symmetry directions of the 2D Brillouin zone. Zero of energy is set at the Fermi level shown by black-dashed line.

Figure 7 shows the electronic energy band diagrams for the different defect configurations. We restrict our analysis to calculations at the PBE level due to computational cost associated with the super cell and the hybrid HSE06 functional. The total (TDOS) and atomic-orbital projected (PDOS) electronic density of states are presented in the Supporting Information. For all structures, the main contribution to the electronic states in the vicinity of Fermi energy level comes from *d*-orbitals of W and *p*-orbitals of Te.

Table 3 summarizes the calculated gaps. The diagonal Te vacancy, the Te(1) vacancy, and the Te(1)-$\hbox {O}_2$ substitution with vertical orientation cause the CBM to move deeper in energy imparting metallic characteristics to the structure. All remaining defects open a small positive band gap at the Fermi level. The antisite defects generally cause an enhanced opening of the gap along the $\Gamma$-X direction, while at the same time moving both conduction and valence states close to the Fermi level near S. A similar behavior is observed for the Te(2)-$\hbox {O}_2$ substitution with parallel orientation. By contrast, a full gap of 58 meV throughout the Brillouin zone is obtained for the parallel Te(1)-$\hbox {O}_2$ defect, which can be explained by the local oxygen bonding to the tungsten atoms. It is worth noting that the electronic structure of both Te(1)-$\hbox {O}_2$ and Te(2)-$\hbox {O}_2$ in vertical orientation resembles closely the one of pristine $\hbox {WTe}_2$. This can be understood by a passivation effect of the local $\hbox {O}_2$ substitution at the vacancy site, similar to what has been observed for substitutional incorporation of atomic oxygen in semiconducting TMD monolayers^60^.

**Figure 8:**
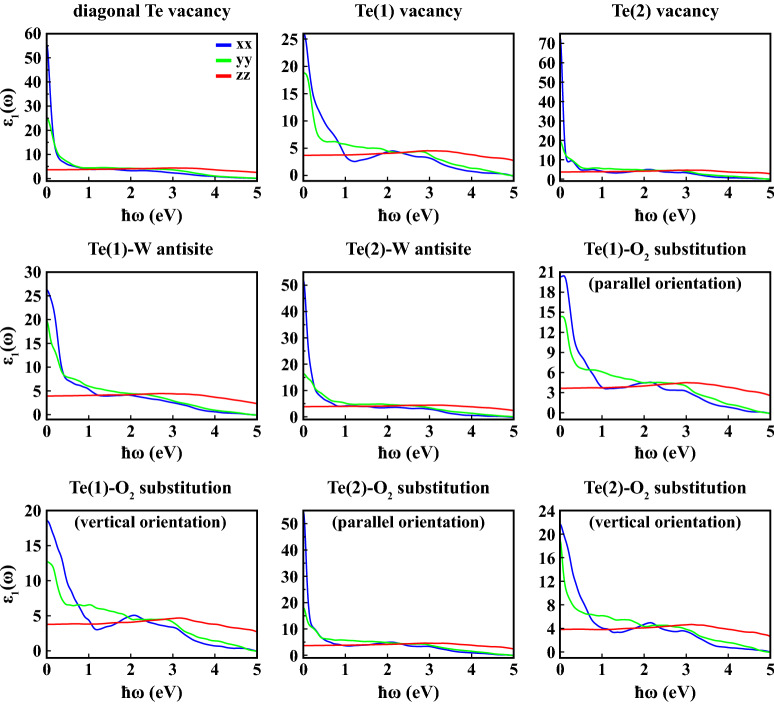
Real part of frequency dependent dielectric constant ($\varepsilon _1(\omega )$) as a function of photon energy ($\hbar \omega$) calculated within PBE+SOC at 0 K along crystallographic axes (*xx*, *yy*, *zz*) for 1L defective 1T′ $\hbox {WTe}_2$.

**Figure 9:**
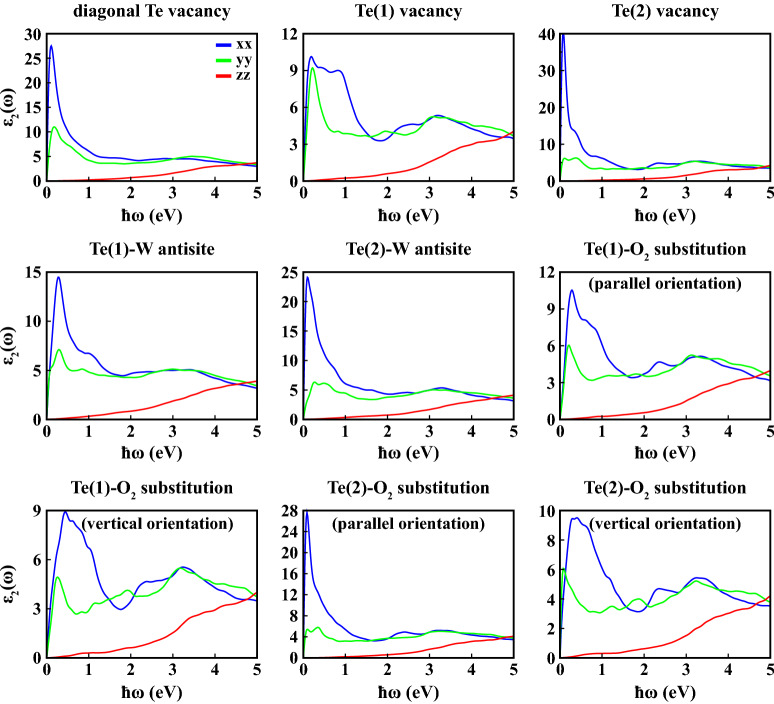
Imaginary part of frequency dependent dielectric constant ($\varepsilon _2(\omega )$) as a function of photon energy ($\hbar \omega$) calculated within PBE+SOC at 0 K along crystallographic axes (*xx*, *yy*, *zz*) for 1L defective 1T′ $\hbox {WTe}_2$.

Figures 8 and 9 display the real ($\varepsilon _1(\omega )$) and imaginary ($\varepsilon _2(\omega )$) parts of the complex dielectric constant for all defective structures.

**Figure 10:**
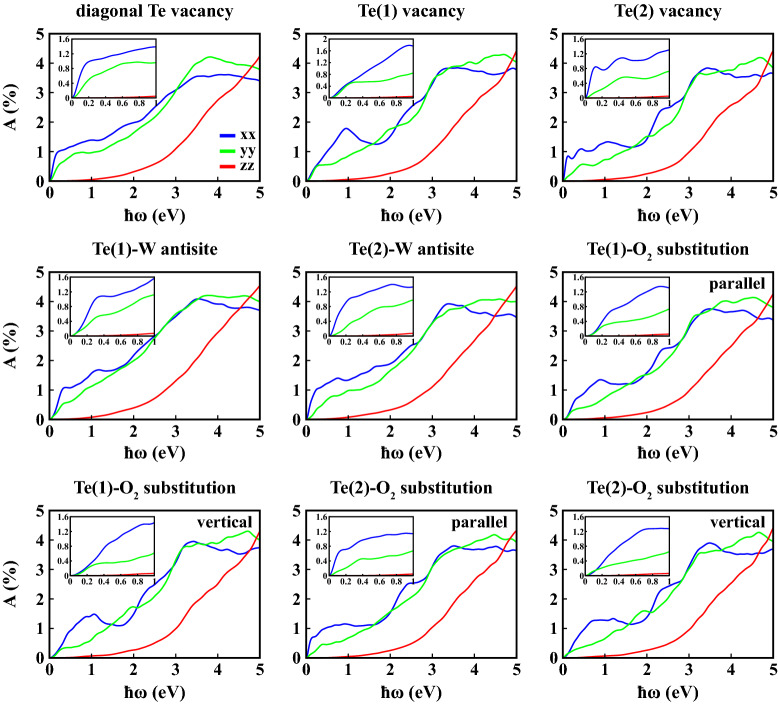
Absorbance (*A*) as a function of photon energy ($\hbar \omega$) calculated within PBE+SOC at 0 K along crystallographic axes (*xx*, *yy*, *zz*) for 1L defective 1T′ $\hbox {WTe}_2$.

We calculated the static dielectric constants for 1L 1T′ $\hbox {WTe}_2$ along *xx* direction (i.e. $\varepsilon _1^{xx}(\omega )$) under the effect of point defects as 55.6, 26.1, 73.4, 26.2, 51.9, 20.3, 18.6, 54.8 and 21.7 for diagonal Te vacancy, Te(1) vacancy, Te(2) vacancy, Te(1)-W antisite, Te(2)-W antisite, Te(1)-$\hbox {O}_2$ substitution with parallel orientation, Te(1)-$\hbox {O}_2$ substitution with vertical orientation, Te(2)-$\hbox {O}_2$ substitution with parallel orientation, Te(2)-$\hbox {O}_2$ substitution with vertical orientation, respectively. All of these values are enhanced compared to pristine $\hbox {WTe}_2$. As for the $\varepsilon _2(\omega )$, in most cases general characteristic preserves itself in the *yy* and *zz* directions. However, in the *xx* lattice direction, while 1L 1T′ $\hbox {WTe}_2$ has two main peaks in the range of 0–2 eV in equilibrium, one peak was obtained with varying intensities for defective structures. The intensities and locations of these main peaks of $\varepsilon _2^{xx}(\omega )$ are calculated 27.5 at 0.110 eV for diagonal Te vacancy, 10.1 at 0.193 eV for Te(1) vacancy, 40.5 at 0.082 eV for Te(2) vacancy, 14.5 at 0.276 eV for Te(1)-W antisite, 24.1 at ${0.097}{\hbox {eV}}$ for Te(2)-W antisite, 10.5 at 0.285 eV for Te(1)-$\hbox {O}_2$ substitution with parallel orientation, 8.9 at 0.438 eV for Te(1)-$\hbox {O}_2$ substitution with vertical orientation, 27.6 at 0.087 eV for Te(2)-$\hbox {O}_2$ substitution with parallel orientation, 9.5 at 0.409 eV for Te(2)-$\hbox {O}_2$ substitution with vertical orientation, respectively. It is clear that the maximum peak in the $\varepsilon _2^{xx}(\omega )$ has been obtained in the Te(2)-vacancy-defective structure. Absorbance of 1L 1T′ $\hbox {WTe}_2$, on the other hand, has not been significantly affected by the point defects considered, both in terms of general trend and intensity (Fig. 10).

**Figure 11:**
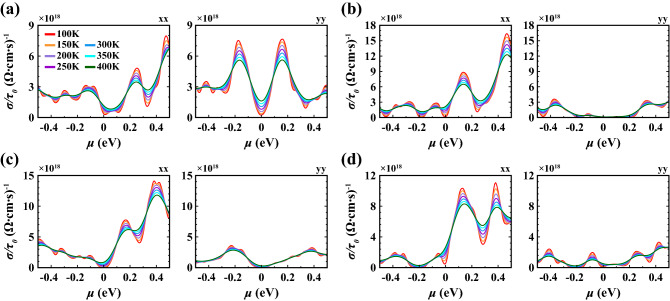
Electrical conductivity with respect to relaxation time ($\sigma /\tau _0$) calculated as a function of chemical potential ($\mu$) at various temperatures along *xx* and *yy* lattice directions for (**a**) Te(1) vacancy, (**b**) Te(2) vacancy, (**c**) Te(1)-$\hbox {O}_2$ substitution with parallel orientation, (**d**) Te(2)-$\hbox {O}_2$ substitution with parallel orientation.

**Figure 12:**
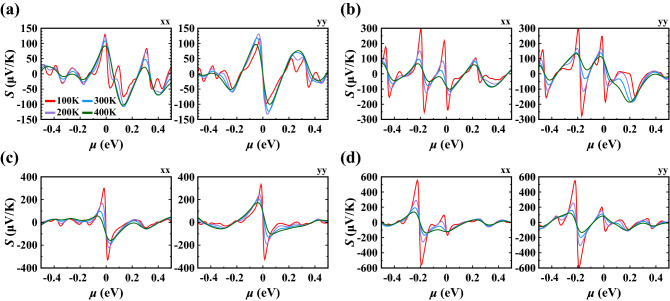
Seebeck coefficient (*S*) calculated as a function of chemical potential ($\mu$) at various temperatures along *xx* and *yy* lattice directions for (**a**) Te(1) vacancy, (**b**) Te(2) vacancy, (**c**) Te(1)-$\hbox {O}_2$ substitution with parallel orientation, (**d**) Te(2)-$\hbox {O}_2$ substitution with parallel orientation.

Lastly, we evaluated the electrical conductivity (Fig. 11) and Seebeck coefficient (Fig. 12). Here, we focused on the Te(1) vacancy and Te(2) vacancy, because they are the simplest point defects relevant in typical experimental settings, as well as on the Te(1)-$\hbox {O}_2$ substitution and Te(2)-$\hbox {O}_2$ substitution with parallel orientation, because they have negative energy of formation and may form under realistic experimental conditions. Results for the other configurations are given in the Supporting Information. While the structure with Te(1) vacancy remains metallic at all temperatures (Fig. 11a), the structure with Te(2) vacancy shows insulating behavior around $\mu = 0$, $\mu = {-0.18}\,{\hbox {eV}}$, $\mu = {-0.45}\,{\hbox {eV}}$ due to the opening of small gaps (Fig. 11b). Furthermore, the conductivity has highly anisotropic properties when comparing *xx* and *yy* lattice directions. The conductivity along *yy* is zero in a wide range around 0 eV which can be related to the directional band gap along $\Gamma$-*Y*. For the Te(1)-$\hbox {O}_2$ substitution (Fig. 11c), we find insulating behaviour at $\mu = 0$, again consistent with the opening of a small positive gap. For the Te(2)-$\hbox {O}_2$ substitution (Fig. 11d), insulating behavior is found for strong hole doping around $\mu ={-0.2}\,\hbox {eV}$ consistent with the corresponding gap observed in the band structure (cf. Fig. 7). These changes in the electronic structure are also reflected in the behavior of the Seebeck coefficient, where a pronounced enhancement of *S* is observed for chemical potentials close to the small electronic gaps induced by the defect modification (Fig. 12). As expected, *S* exhibits also a sign change close to these values. We note that the absolute value of the Seebeck coefficient can potentially be enhanced via defect modification and doping, e.g. to ${600}\,\upmu \hbox {V}\,\hbox {K}^{-1}$ for the Te(2)-$\hbox {O}_2$ substitution in Fig. 12.

## Discussion

In this study, we explored the effects of layer thickness and defects such as vacancy, antisite, and substitution on the electronic, optical and thermoelectric properties of 1T′ $\hbox {WTe}_2$. We showed that by going from a single layer to four layers the fundamental band gap is decreased and probably closed in multilayer tungsten ditelluride. These changes are also reflected in the optical properties, where we found a significant modification of the dielectric constant below approximately ${1}\,\hbox {eV}$. The number of the layers also leads to changes in the thermoelectric properties. While the thermopower (*S*) decreases with increasing number of layers, the conductivity ($\sigma /\tau _0$) is raised. Overall, the anisotropic crystal structures of $\hbox {WTe}_2$ manifests in anisotropic electronic properties, whereby our calculations demonstrate that the anisotropy is most pronounced for strong electron doping.

Beyond layer tuning, the creation of point defects offers new features for monolayer 1T′ $\hbox {WTe}_2$. Under point defects, the energy band gaps are affected in a significant manner. While the diagonal Te vacancy, the Te(1) vacancy, and the Te(1)-$\hbox {O}_2$ substitution with vertical orientation defective structures are metallic with remarkable electronic density of states at $\hbox {E}_F$, the other studied point defects open the narrow band gaps in the electronic spectrum. We correlate these changes in the electronic spectrum to the corresponding changes in the optical and thermoelectric properties. The imaginary part of the dielectric constant of 1T′ $\hbox {WTe}_2$ can alter in the *xx* lattice direction in such a way that while it has two major peaks in the range of 0–2 eV at equilibrium, one peak of varying intensities was realized for defective structures. Besides, we obtained an enhancement in the Seebeck coefficient of 1T′ $\hbox {WTe}_2$ for the chemical potential values close to the small electronic band gaps induced by the defect modification. Particularly, the absolute value of the value of *S* can potentially be enhanced up to ${600}\,\upmu \hbox {V}\,\hbox {K}^{-1}$ for the Te(2)-$\hbox {O}_2$ substitution. Since the Seebeck coefficient of 1T′ $\hbox {WTe}_2$ in equilibrium, which is in the range of 51–113 $\upmu \hbox {V}\,\hbox {K}^{-1}$, below a reasonable value for applications (200 $\upmu \hbox {V}\,\hbox {K}^{-1}$), such an improvement via defect engineering could potentially pave the way for 1T′ $\hbox {WTe}_2$ in thermoelectric devices.

## Methods

Our theoretical analysis, based on spin-polarized density functional theory (DFT), was performed using the Vienna Ab initio Simulation Package (VASP)^61,62^. We used projected augmented wave (PAW) potentials^63,64^ to describe the ion-electron interactions, and proposed generalized gradient approximation (GGA) by using the Perdew–Burke–Ernzerhof (PBE)^65^ functional for the electronic exchange-correlation potential. To include van der Waals interactions, the method of Grimme (DFT-D2) was employed^66^. The energy cutoff for the plane wave basis was set to $\hbar ^2({{\textbf {k}}}+{{\textbf {G}}})^2/2m={500}$ eV. For the Brillouin zone (BZ) integration in **k**-space, a set of ($18\times 10\times 1$) **k**-points were used within the Monkhorst–Pack scheme^67^. Spin-orbit coupling (SOC) was included in all calculations. The structures were fully optimized by using the conjugate gradient algorithm^68,69^ until the Hellmann–Feynman force on each atom was less than ${0.01}\,{\hbox {eV}}/{\text{\AA} }$ and the maximum pressure in the unit cell was below ${0.5}\,\hbox {kbar}$. We visualized all structures by the VESTA program^70^. We also performed hybrid functional (HSE06) calculations, which is known to predict the electronic structure more accurately compared to the PBE results^46^. The screening length of HSE06 was taken as ${0.2}{\text{\AA} }$ and the mixing rate of the Hartree–Fock exchange potential was set to 0.25. Further details of our calculations are presented in the Supporting Information.

The cohesive energies per atom $E_{\text {coh}}$ and formation energies of the point defects $E_{\text {for}}$ were calculated by the following equations:

1 $$\begin{aligned} E_{\text {coh}}= & {} \frac{[(k\times E_{\text {W}})+(l\times E_{\text {Te}})+(m\times E_{\text {O}})]-E_{\text {total}}}{k+l+m} \end{aligned}$$

2 $$\begin{aligned} E_{\text {for}}= & {} E_{\text {def}}-E_{\text {pure}}+\mu _{\text {sub}}-\mu _{\text {add}} \end{aligned}$$

In Eq. (1), *k*, *l* and *m* indicate the number of corresponding atoms in the cell. $E_{\text {W}}$, $E_{\text {Te}}$, $E_{\text {O}}$ and $E_{\text {total}}$ represent the total energies of W, Te and O single atoms and of the $\hbox {WTe}_2$ system, respectively. In Eq. (2), $E_{\text {def}}$ and $\hbox {E}_{\text {pure}}$ stand for the total energies of specified defective and pristine structures of $\hbox {WTe}_2$, respectively. $\mu _{\text {sub}}$ and $\mu _{\text {add}}$ correspond to the chemical potentials of subtracted and added atoms. We obtained the chemical potentials of W and Te from body-centered cubic and trigonal crystal structures known as their most stable forms. The chemical potential of O was derived from $\hbox {O}_2$ gas, which is the most stable form of oxygen. Chemical potentials of W and Te, $\mu _{\text {W}}$ and $\mu _{\text {Te}}$, satisfy the relation $\mu _{\mathrm{W}}+2\mu _{\mathrm{Te}}=\mu _{{\mathrm{WTe}}_2}$. In Te-rich environment, we used $\mu _{\text {Te}}$ as its own value and derived $\mu _{\text {W}}$ from the chemical potential relation just described and vice versa for W-rich environment.

To investigate the optical properties, we calculated the imaginary part of the dielectric constant ($\varepsilon _2$) by a summation of all possible transitions from occupied to unoccupied states using the following equation:

3 $$\begin{aligned} \varepsilon _2^{\alpha \beta }(\omega )=\frac{4\pi ^2e^2}{\Omega }\lim _{q\rightarrow 0}\frac{1}{q^2}\sum _{c,v,{{\textbf {k}}}}2w_{{\textbf {k}}}\delta (\varepsilon _{c{{\textbf {k}}}}-\varepsilon _{v{{\textbf {k}}}}-\omega ) \times \langle u_{c{{\textbf {k}}}+e_{\alpha } {{\textbf {q}}}}|u_{v{{\textbf {k}}}}\rangle \langle u_{c{{\textbf {k}}}+e_{\beta } {{\textbf {q}}}}|u_{v{{\textbf {k}}}}\rangle ^* \end{aligned}$$

Here, *c* and *v* denote the conduction and valence band states, and $u_{c{{\textbf {k}}}}$ represents the cell periodic part of the wave function at the **k**-point. The real part of the dielectric constant ($\varepsilon _1$) was obtained by the Kramers–Kronig transformation^71^ as follows:

4 $$\begin{aligned} \varepsilon _1^{\alpha \beta }(\omega )=1+\frac{2}{\pi }P\int ^{\infty }_{0}\frac{\varepsilon _2^{\alpha \beta }(\omega ')\omega '}{\omega '^2-\omega ^2+i\eta }d\omega ' \end{aligned}$$

The total frequency dependent complex dielectric constant is then the sum of these two terms as $\varepsilon (\omega )=\varepsilon _1(\omega )+i\varepsilon _2(\omega )$. With the frequency dependent complex dielectric constant, we calculated the absorbance (*A*) according to:^72^

5 $$\begin{aligned} A(\omega )=\frac{\omega }{c}\varepsilon _2(\omega )\Delta z \end{aligned}$$

Here, $\omega$ is the photon angular frequency, *c* is the speed of light and $\Delta z$ is the thickness of the crystal slab.

Anisotropic thermoelectric transport coefficients of monolayer and multilayer $\hbox {WTe}_2$, specifically the Seebeck coefficient (*S*) and electrical conductivity with respect to relaxation time ($\sigma /\tau _0$), have been obtained by the BoltzTraP2 code^52^ in conjunction with PBE results using an interpolated, 3-times denser **k**-mesh. BoltzTraP2 calculates the transport coefficients by solving the semi-classical Boltzmann transport equation within the rigid-band approximation (RBA), which assumes that changing the temperature, or doping a system, does not change the band structure, in combination with the constant relaxation time approximation (CRTA), which means that the Seebeck coefficient becomes independent of the scattering rate^52,73^. Under CRTA, the generalized transport coefficients are obtained by the following equation:

6 $$\begin{aligned} {\mathscr {L}}^{(\alpha )}(\mu ;T)=q^2 \int \sigma (\varepsilon ,T)(\varepsilon -\mu )^{\alpha } \Bigg ( -\frac{\partial \textit{f}^{(0)}(\varepsilon ;\mu ,T)}{\partial \varepsilon } \Bigg ) \end{aligned}$$

Herein, $\sigma (\varepsilon ,T)$ is the transport distribution function obtained by interpolation of the electronic band structure and given by

7 $$\begin{aligned} \sigma (\varepsilon ,T)=\int \sum _b {{\textbf {v}}}_{b,{{\textbf {k}}}} \otimes {{\textbf {v}}}_{b,{{\textbf {k}}}}~ \tau _{b,{{\textbf {k}}}}~ \delta (\varepsilon -\varepsilon _{b,{{\textbf {k}}}})~ \frac{d{{\textbf {k}}}}{8\pi ^3} \end{aligned}$$

where $\varepsilon _{b,{{\textbf {k}}}}$ and ${{\textbf {v}}}_{b,{{\textbf {k}}}}$ are the energy and velocity of an electron situated at the corresponding band in the wavevector **k**, $\tau$ denotes the relaxation time, and the integral is taken over the whole Brillouin zone. Thus, Seebeck coefficient (*S*) and electrical conductivity ($\sigma$) are calculated as follows:

8a $$\begin{aligned} S&=\frac{1}{qT}\frac{{\mathscr {L}}^{(1)}}{{\mathscr {L}}^{(0)}} \end{aligned}$$

8b $$\begin{aligned} \sigma&={\mathscr {L}}^{(0)} \end{aligned}$$

## Supplementary Information

Supplementary Information.

## Data Availability

All data generated or analysed during this study are included in this published article (and its Supplementary Information files). Results of refractive index calculations are available in the zenodo repository, [https://zenodo.org/record/6875279#.YtlX1YTP1eU].
