# Supplementary material for: Thickness and defect dependent electronic, optical and thermoelectric features of WTe2
Source: Sci Rep. 2022 Jul 26;12:12756. doi: 10.1038/s41598-022-16899-5 (PMC9325696; doi:10.1038/s41598-022-16899-5)
Supplement: Supplementary file 1 — Supplementary Information. [file 41598_2022_16899_MOESM1_ESM.pdf]

Supporting Information for Thickness and Defect Dependent Electronic, Optical and Thermoelectric Features of WTe_2

Ilkay Ozdemir[1], Christoph Kastl[2,3], Alexander W. Holleitner[2,3], and Olcay Üzengi Aktürk[2,4,*]

[1]Physics Department, Adnan Menderes University, Aydin 09100, Turkey
[2]Walter Schottky Institut and Physics Department, Technical University of Munich, Am Coulombwall 4a, 85748 Garching, Germany
[3]Munich Center of Quantum Science and Technology (MCQST), Schellingstr. 4, 80799 Munich, Germany
[4]Electrical Electronics Engineering Department, Adnan Menderes University, Aydin 09100, Turkey
[*]ouzengi@adu.edu.tr

ABSTRACT

Transition metal dichalcogenides (TMDs) receive significant attention due to their outstanding electronic and optical properties. In this study, we investigate the electronic, optical, and thermoelectric properties of single and few layer WTe_2 in detail utilizing first-principles methods based on the density functional theory (DFT). Within the scope of both PBE and HSE06 including spin orbit coupling (SOC), the simulations predict the electronic band gap values to decrease as the number of layers increases. Moreover, spin-polarized DFT calculations combined with the semi-classical Boltzmann transport theory are applied to estimate the anisotropic thermoelectric power factor (Seebeck coefficient, S) for WTe_2 in both the monolayer and multilayer limit, and S is obtained below the optimal value for practical applications. The optical absorbance of WTe_2 monolayer is obtained to be slightly less than the values reported in literature for 2H TMD monolayers of MoS_2, $MoSe_2$, and WS_2. Furthermore, we simulated~~d~~ the impact of defects, such as vacancy, antisite and substitution defects, on the electronic, optical and thermoelectric properties of monolayer WTe_2. Particularly, the Te-O_2 substitution defect in parallel orientation yields negative formation energy, indicating that the relevant defect may form spontaneously under relevant experimental conditions. We reveal that the electronic band structure~~s~~ of WTe_2 monolayer is significantly influenced by the presence of ~~point~~ the considered defects. According to the calculated band gap values, a lowering of the conduction band minimum gives rise to metallic characteristics to the structure for the single Te(1) vacancy, a diagonal Te line defect, and the Te(1)-O_2 substitution, while the other investigated defects cause an opening of a small positive band gap at the Fermi level. ~~Furthermore~~Consequently, the real $(\varepsilon_1(\omega))$ and imaginary $(\varepsilon_2(\omega))$ parts of the dielectric constant at low frequencies ~~values~~ are very sensitive to the applied ~~point~~ defects, whereas we find that the absorbance (A) ~~of WTe_2 is not significantly affected~~ at optical frequencies is less significantly affected. We also predict that certain point defects can enhance the otherwise moderate value of S in pristine WTe_2 to values relevant for thermoelectric applications. The described WTe_2 monolayers, as functionalized with ~~point~~ the considered defects, offer the possibility to be applied in optical, electronic, and thermoelectic devices.

Section S1: Electronic structures of monolayer and multilayer 1T' WTe_2

Here, we present electronic energy band structures, $(E_n(\mathbf{k}))$, of monolayer and multilayer 1T' WTe_2 calculated within PBE and HSE06 functionals without and with spin-orbit coupling (SOC) parameter along major symmetry directions of two dimensional (2D) Brillouin zone (BZ), that is, Γ(0,0,0)-X(0.5,0,0)-S(0.5,0.5,0)-Y(0,0.5,0)-Γ(0,0,0). In addition to the 1L, 2L, 3L and 4L structures, we further calculated the band structures of 5L and 6L WTe_2 (Figure 6) with PBE for comparison purposes, band gaps of which follow the same trend as the former ones as described in the main text. We depicted the band gap values calculated with PBE for 1L-to-6L WTe_2 and with HSE06 for 1L-to-4L WTe_2 in Figure 1, below. (We could not perform HSE06 calculations for 5L and 6L WTe_2 due to computational cost.)

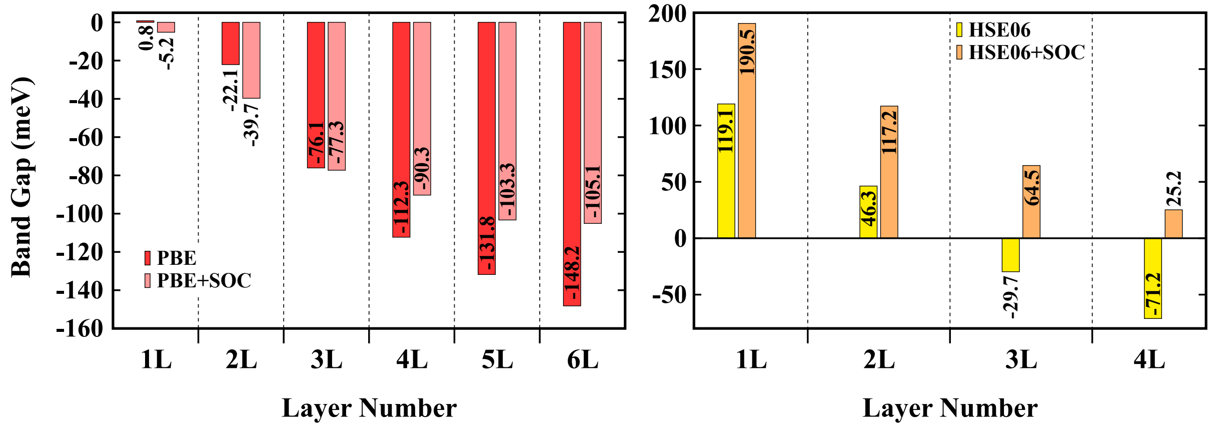

Figure 1. Layer dependence of the band gap values of monolayer and multilayer 1T' WTe_2 calculated by PBE and HSE06 functionals without and with SOC.

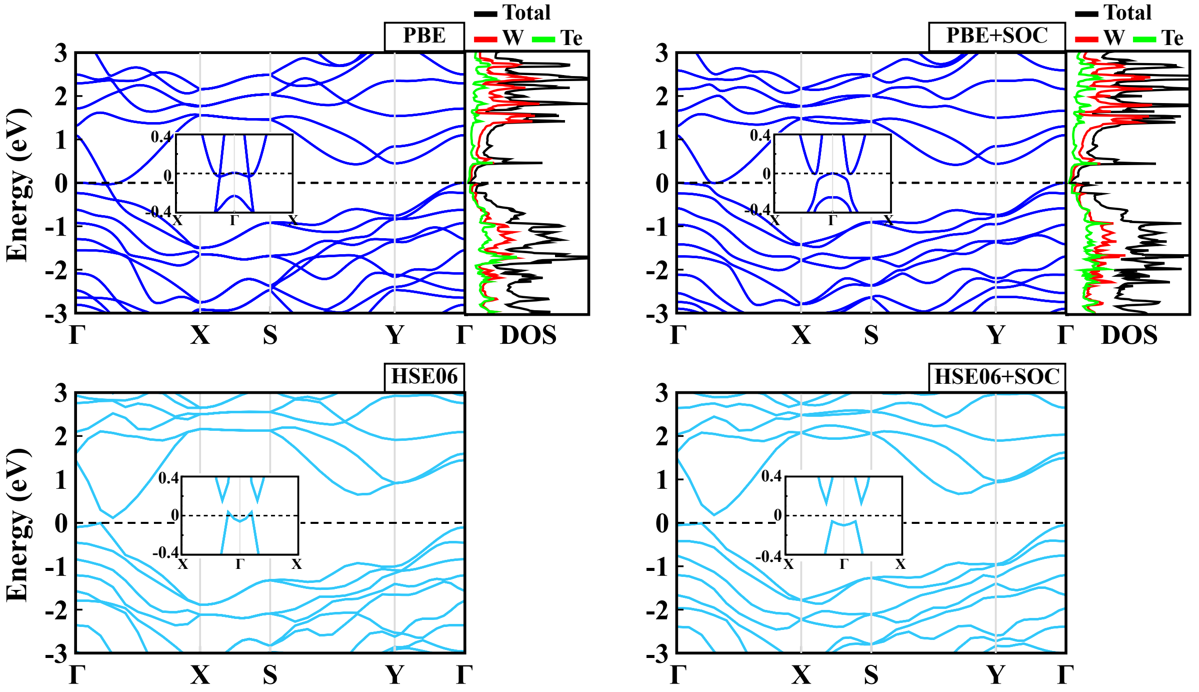

Figure 2. Electronic energy band structures, $E_n(\mathbf{k})$, calculated for 1L 1T' WTe_2 within PBE and HSE06 functionals without and with SOC along major symmetry directions of the 2D BZ and related PDOS. Zero of energy is set at the Fermi level shown by black-dashed line. The region in the vicinity of indirect band gap lying between Γ-X points is highlighted as inset.

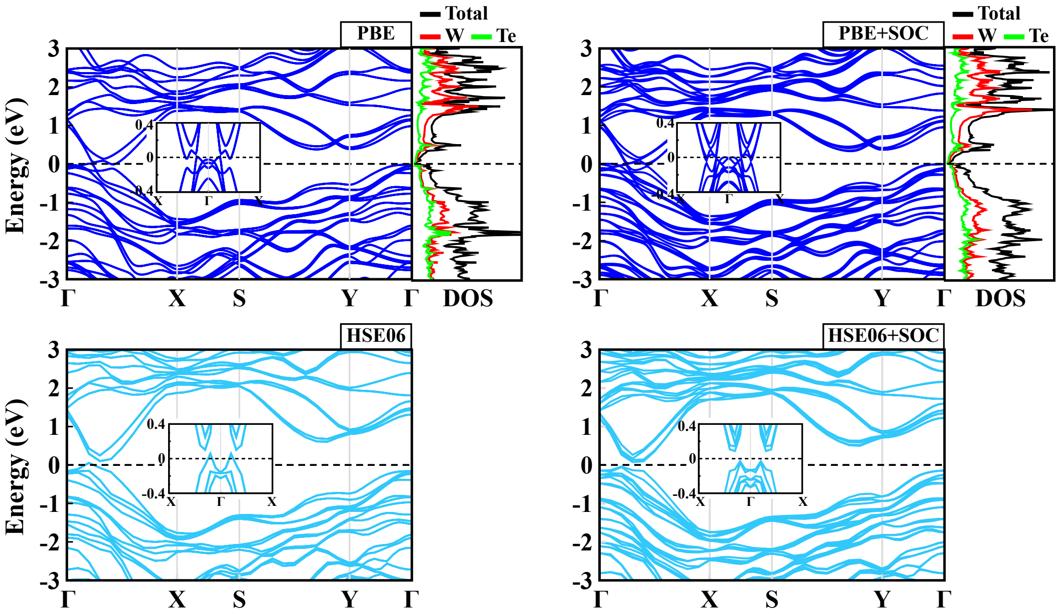

Figure 3. Electronic energy band structures, $E_n(\mathbf{k})$, calculated for 2L 1T' WTe_2 within PBE and HSE06 functionals without and with SOC along major symmetry directions of the 2D BZ and related PDOS. Zero of energy is set at the Fermi level shown by black-dashed line. The region in the vicinity of indirect band gap lying between Γ-X points is highlighted as inset.

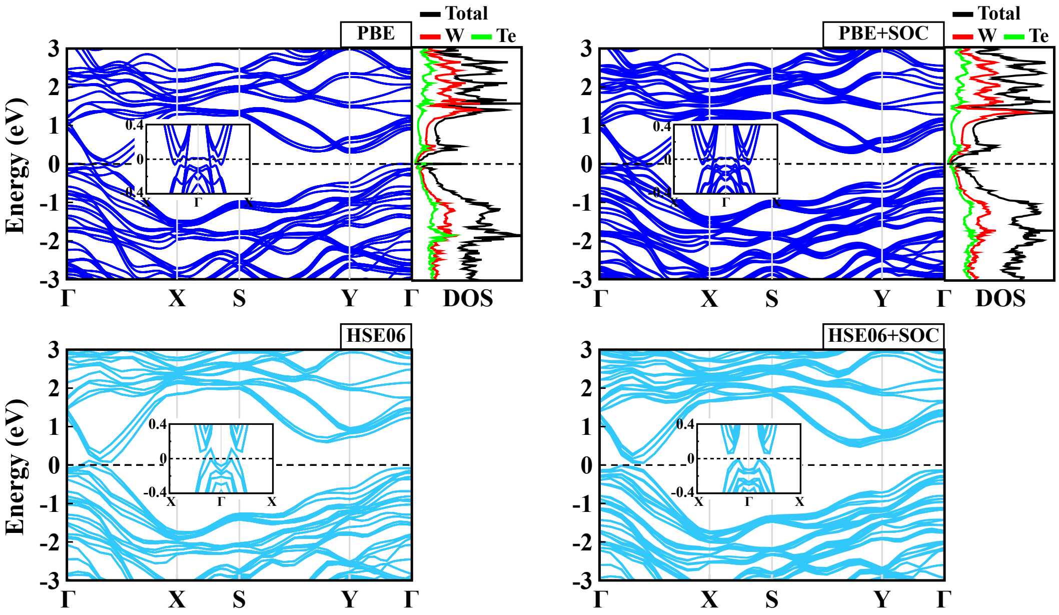

Figure 4. Electronic energy band structures, $E_n(\mathbf{k})$, calculated for 3L 1T' WTe_2 within PBE and HSE06 functionals without and with SOC along major symmetry directions of the 2D BZ and related PDOS. Zero of energy is set at the Fermi level shown by black-dashed line. The region in the vicinity of indirect band gap lying between Γ-X points is highlighted as inset.

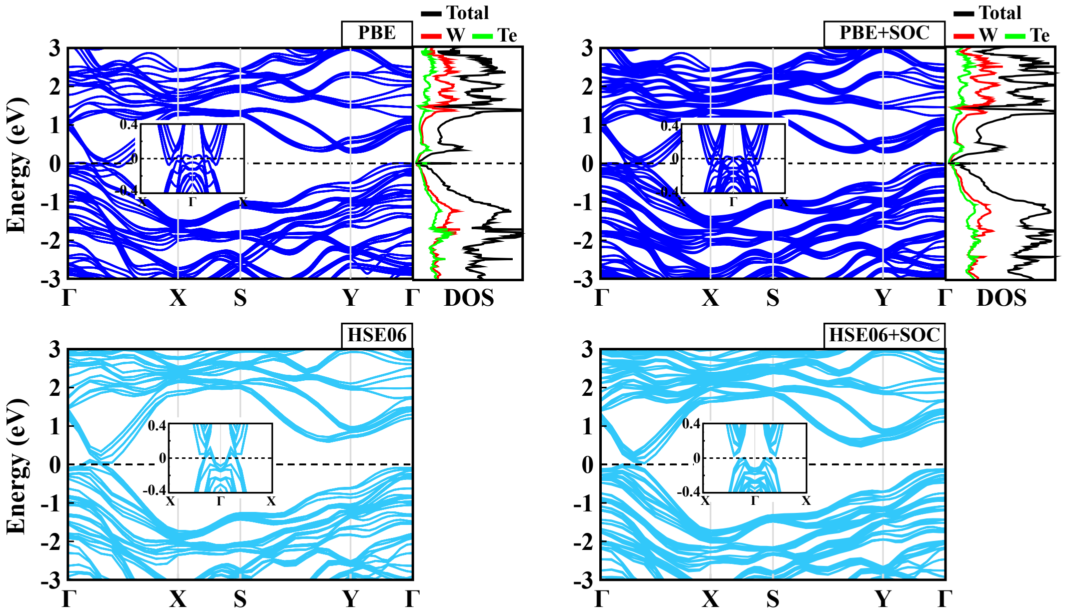

Figure 5. Electronic energy band structures, $E_n(\mathbf{k})$, calculated for 4L 1T' WTe_2 within PBE and HSE06 functionals without and with SOC along major symmetry directions of the 2D BZ and related PDOS. Zero of energy is set at the Fermi level shown by black-dashed line. The region in the vicinity of indirect band gap lying between Γ-X points is highlighted as inset.

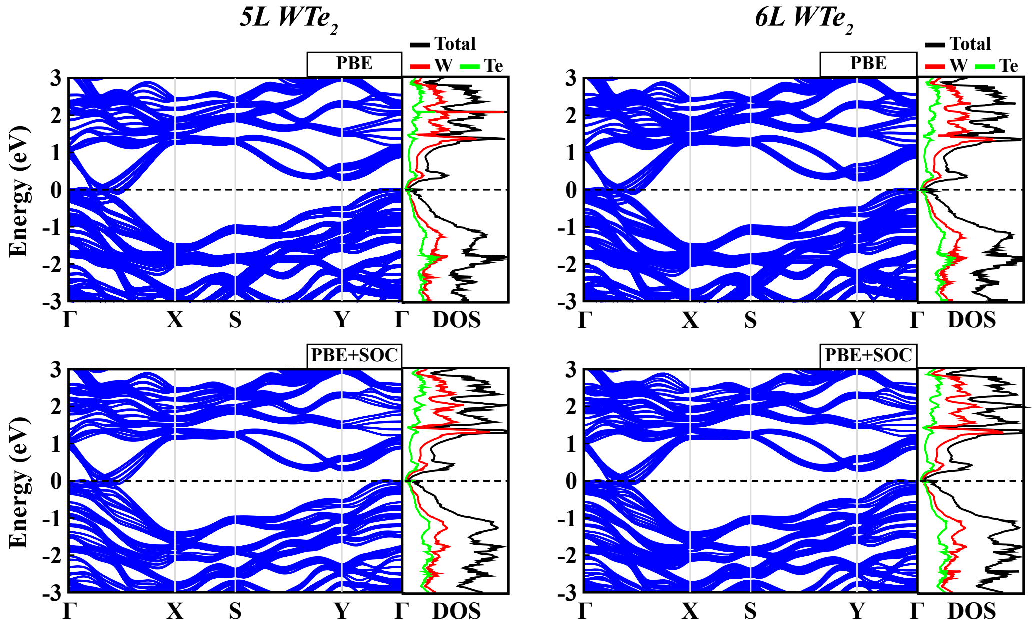

Figure 6. Electronic energy band structures, $E_n(\mathbf{k})$, calculated for 5L and 6L 1T' WTe_2 within PBE functional without and with SOC along major symmetry directions of the 2D BZ and related PDOS. Zero of energy is set at the Fermi level shown by black-dashed line. The region in the vicinity of indirect band gap lying between Γ-X points is highlighted as inset.

Section S2: Optical properties of bilayer and trilayer 1T' WTe_2

Here, we present the optical properties, i.e. real ($\varepsilon_1(\omega)$) and imaginary ($\varepsilon_2(\omega)$) parts of the frequency dependent complex dielectric constant and absorbance (A) of 2L and 3L 1T' WTe_2 as a function of photon energy ($\hbar\omega$). We calculated the optical properties at 0 K along in-plane ($\mathbf{E}\| x$ and $\mathbf{E}\| y$) and out-of-plane ($\mathbf{E}\| z$) directions within the photon energy range of 0-5 eV in PBE level including SOC. (Figure 7)

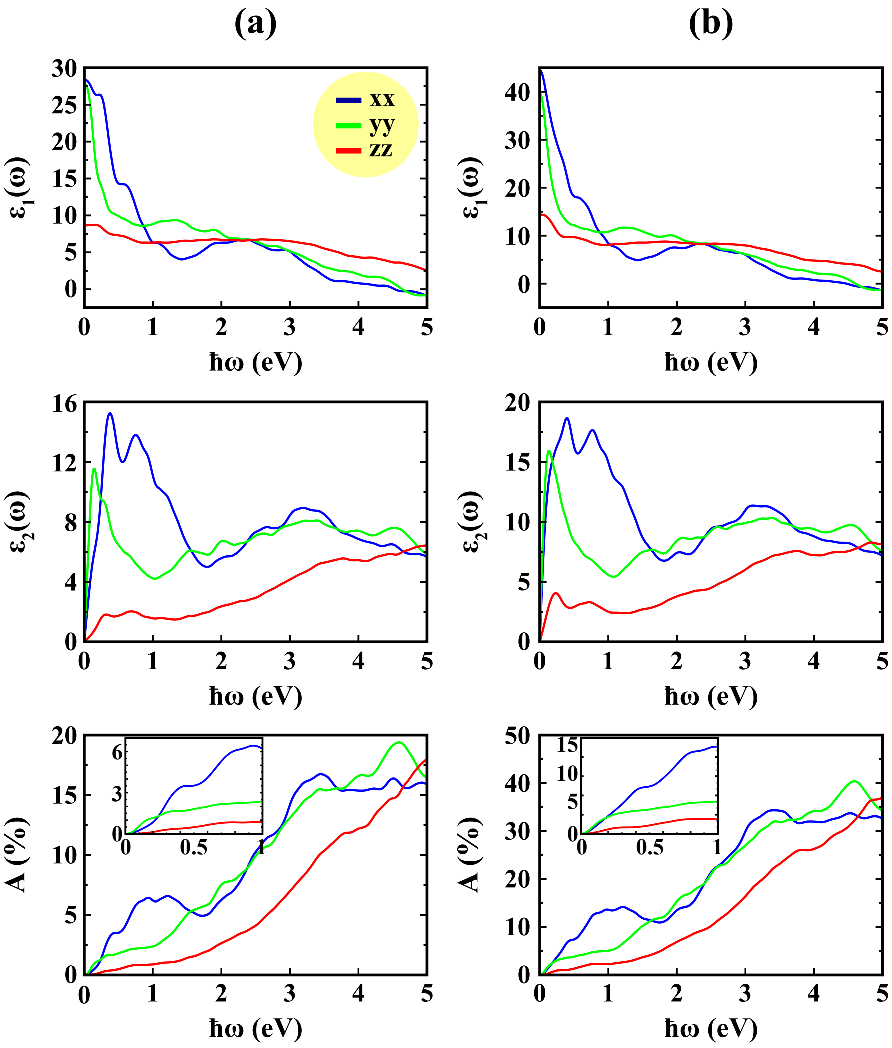

Figure 7. Real ($\varepsilon_1(\omega)$) and imaginary ($\varepsilon_2(\omega)$) parts of frequency dependent dielectric constant, and absorbance (A) calculated within PBE+SOC for **(a)** 2L and **(b)** 3L 1T' WTe_2 as a function of photon energy ($\hbar\omega$) at 0 K along crystallographic axes (*xx*, *yy*, *zz*).

Section S3: Seebeck coefficient of 1T' WTe_2

Here, we present Seebeck coefficients (S) of 2L and 3L 1T' WTe_2 calculated as a function of chemical potential (μ) at various temperatures (T) and as a function of temperature T within the range of 100-400 K for chemical potentials between −0.5 to 0.5 eV along xx and yy lattice directions. (Figure 9) The peak values of the Seebeck coefficient (S) of monolayer and multilayer 1T' WTe_2 obtained at 300 K are summarized in Figure 8. As is known, negative and positive values of S indicate that n-type and p-type carriers are dominant in the structure, respectively. Thus, the S peaks in the Figure 8 maybe interpreted in such a way that, in xx-direction, n-type carriers are dominant in 1L and 2L WTe_2, whereas p-type carriers are dominant in 3L and 4L WTe_2. In yy-direction, on the other hand, while n-type carriers are dominant in 1L WTe_2, p-type carriers are dominant in 2L, 3L and 4L WTe_2.

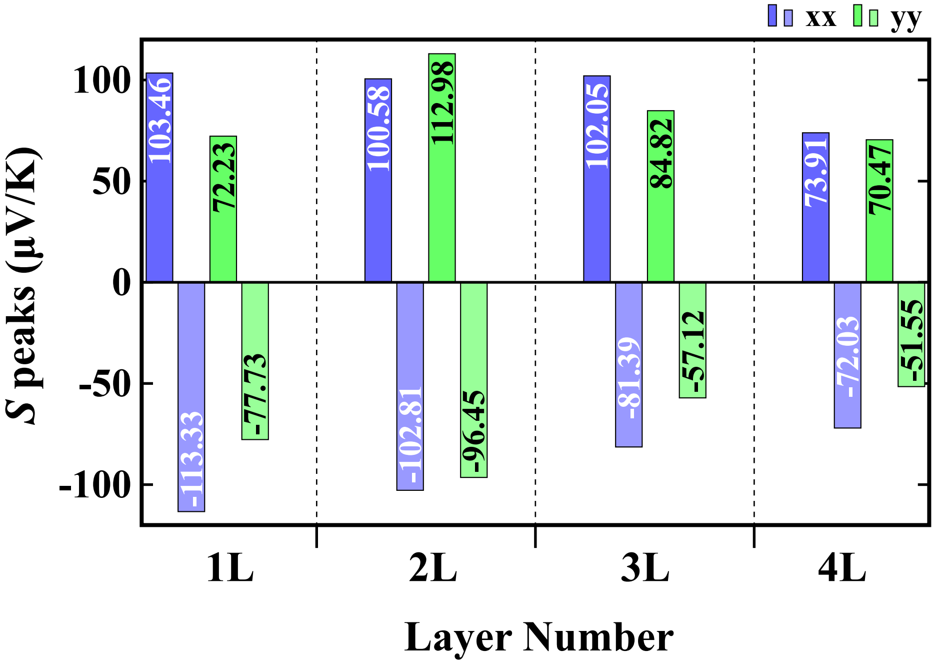

Figure 8. Positive and negative peak values of Seebeck coefficient for 1L, 2L, 3L and 4L 1T' WTe_2 calculated at 300 K.

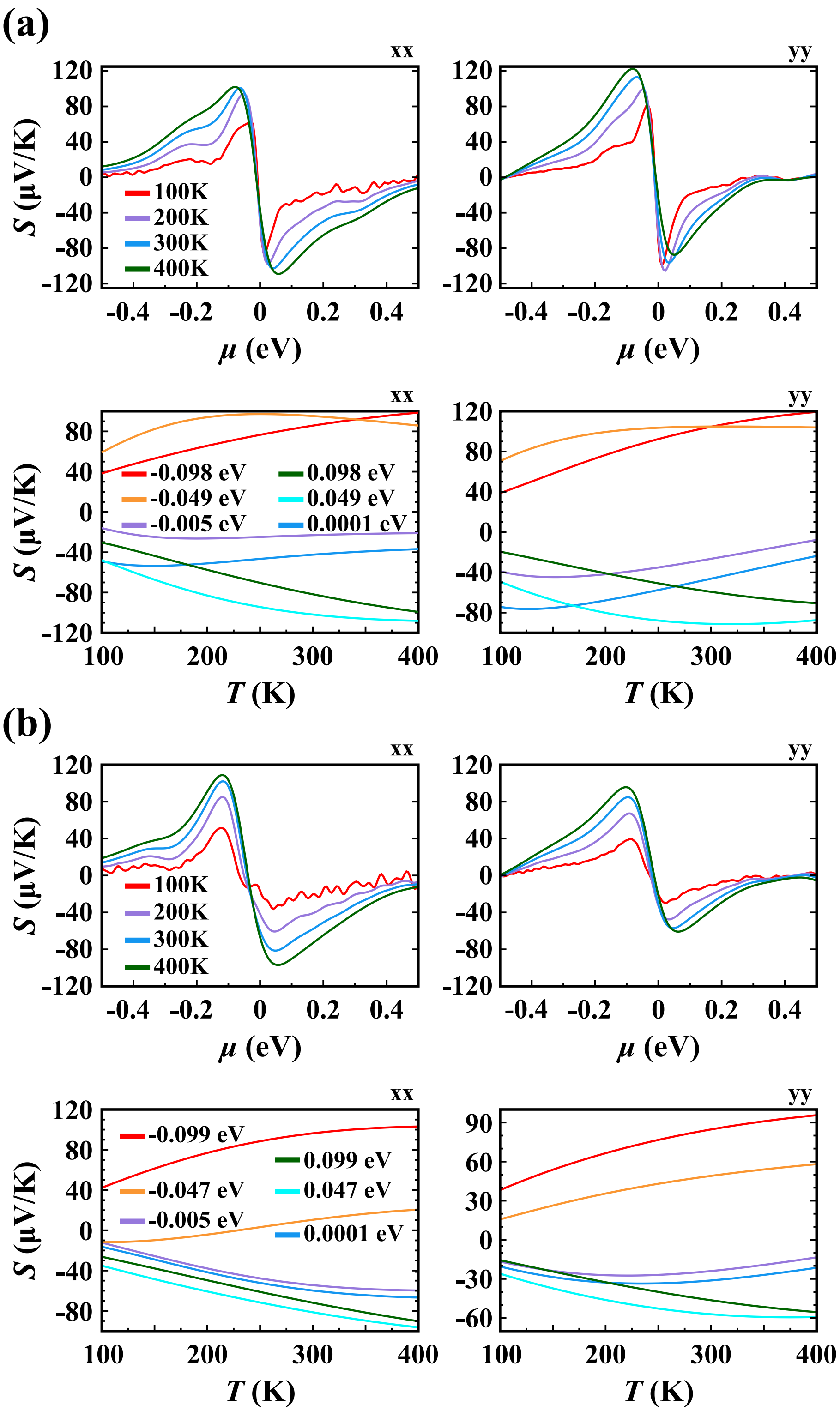

Figure 9. Seebeck coefficient (S) calculated as a function of chemical potential (μ) at various temperatures and as a function of temperature (T) at various chemical potentials for **(a)** 2L and **(b)** 3L 1T' WTe_2 along xx and yy lattice directions.

Section S4: Electrical conductivity of 1T' WTe_2

Here, we present the electrical conductivities with respect to constant relaxation time (σ/τ_0) of 1L, 2L, 3L and 4L 1T' WTe_2 calculated as a function of chemical potential (μ) at 300 K and as a function of temperature (T) within the range of 100-400 K for various chemical potentials. (Figure 10) As can be seen, σ/τ_0 is less sensitive to temperature than S. Besides, conductivity is higher in the *xx* direction for positive μ values (i.e. n-type region), that is, highly anisotropic, while it is almost isotropic for negative μ values (i.e. p-type region).

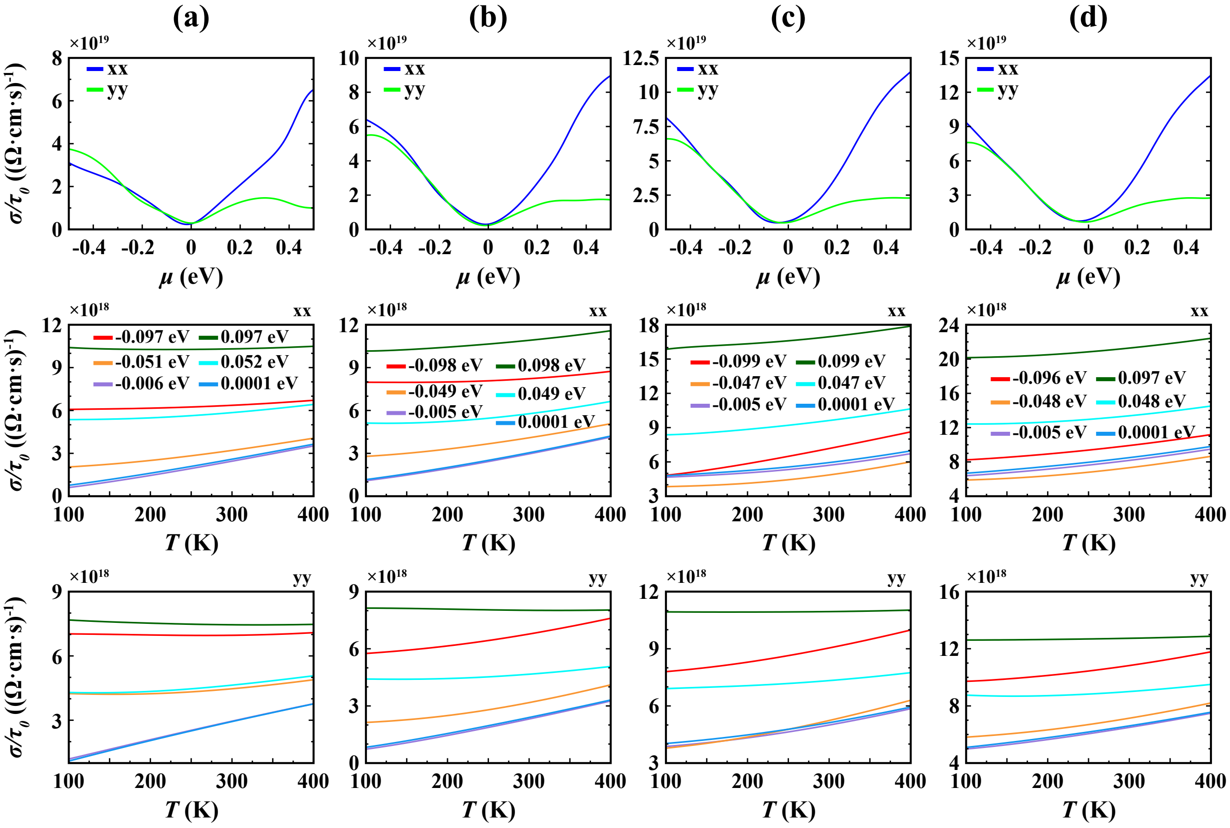

Figure 10. Electrical conductivity with respect to relaxation time (σ/τ_0) calculated as a function of chemical potential (μ) at 300 K and as a function of temperature (T) at various chemical potentials for **(a)** 1L, **(b)** 2L, **(c)** 3L and **(d)** 4L 1T' WTe_2 along *xx* and *yy* lattice directions.

Section S5: Initial atomic structures of defective 1L 1T' WTe_2

Here, we present the initial atomic structures of defective 1L 1T' WTe_2. (Figure 11)

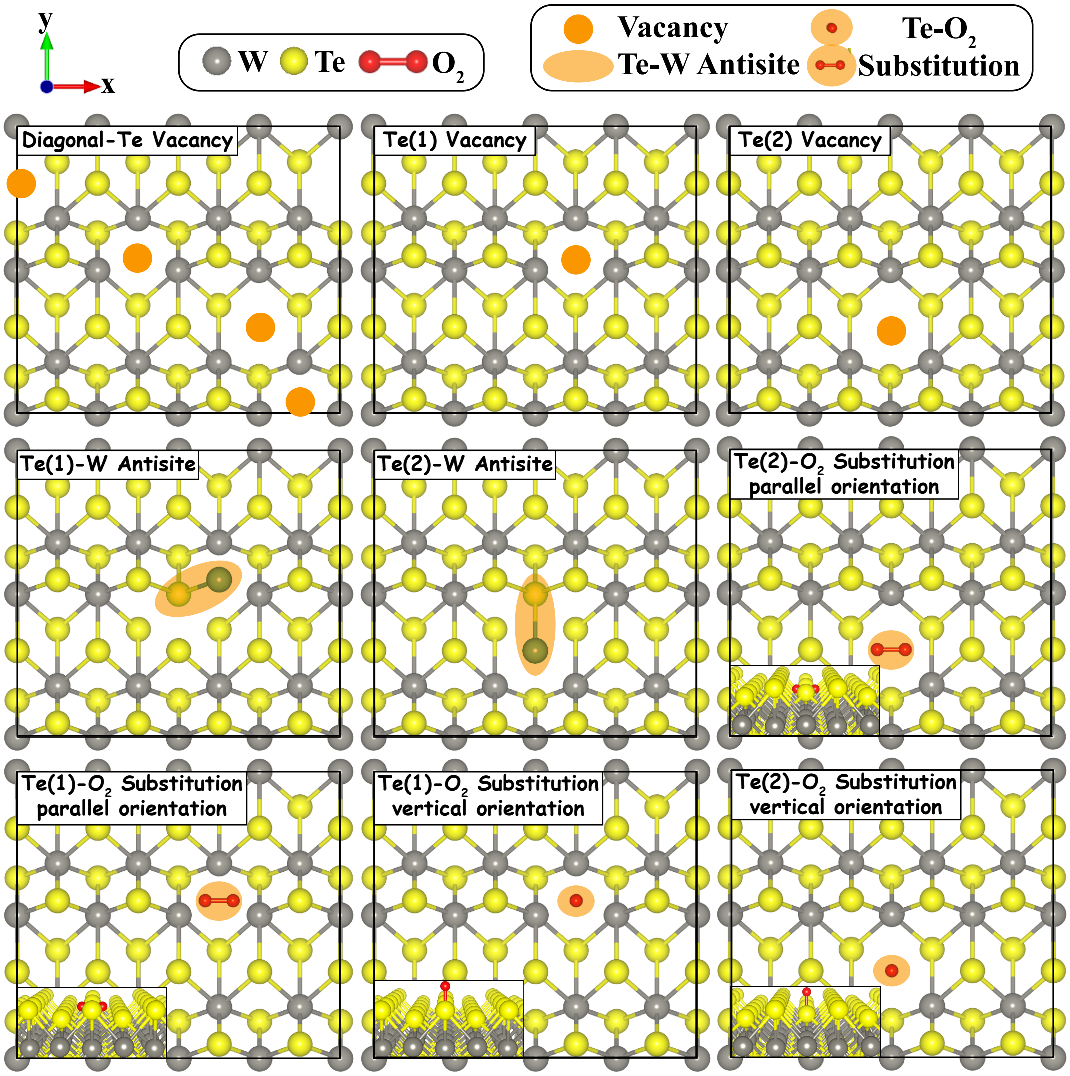

Figure 11. Initial lattice configurations (from top view, i.e. *xy*-plane) of the point defects considered in this study for 1L 1T' WTe_2 within a (4×2) super cell. The insets show side views of the parallel and vertical configurations of the *Te-O*$_2$ substitution geometries.

Section S6: Charge transfer analysis in Te-O_2 substitution defects

Here, we present the isosurfaces of charge density difference ($\Delta\rho(\mathbf{r})$) between Te-vacancy defective WTe_2 surface and O_2 molecule substituted with subtracted Te atom, i.e. Te-O_2 substitution. This was obtained by subtracting the charge densities situated at the surface (Te-vacancy defective WTe_2 surface) and molecular (O_2) sites from the total electronic charge density of the system ($\rho_T(\mathbf{r})$). The resulting isosurfaces reflect electronic charge transfers between the surface and the O_2 molecule. As can be seen from the Fig. 12 (a) and (b), in parallel orientations O_2 molecule is no longer a molecule, but rather has dissociated and each O atom individually bound to the surface. By contrast, in the vertical configurations, the oxygen molecule is not dissociated, but it rather binds to three neighboring W (Te) atoms by means of a local charge transfer creating an O_{2-} complex (Fig. 12 (c) and (d)). Besides, we quantitatively calculated the amount of interatomic electronic charge transfer by Bader charge analysis[1]. The calculated excess charges on the O atoms are given in Table 1.

Table 1. Amount of excess electronic charges residing on O-atomic-sites in the Te-O_2 substitution defective structures calculated by Bader charge analysis.

atomic	Te(1)-O_2 substitution		Te(2)-O_2 substitution	
site	parallel	vertical	parallel	vertical
O(1) (e)	0.8367	-0.1232	1.0863	0.4805
O(2) (e)	0.8325	0.0224	1.0155	0.3719

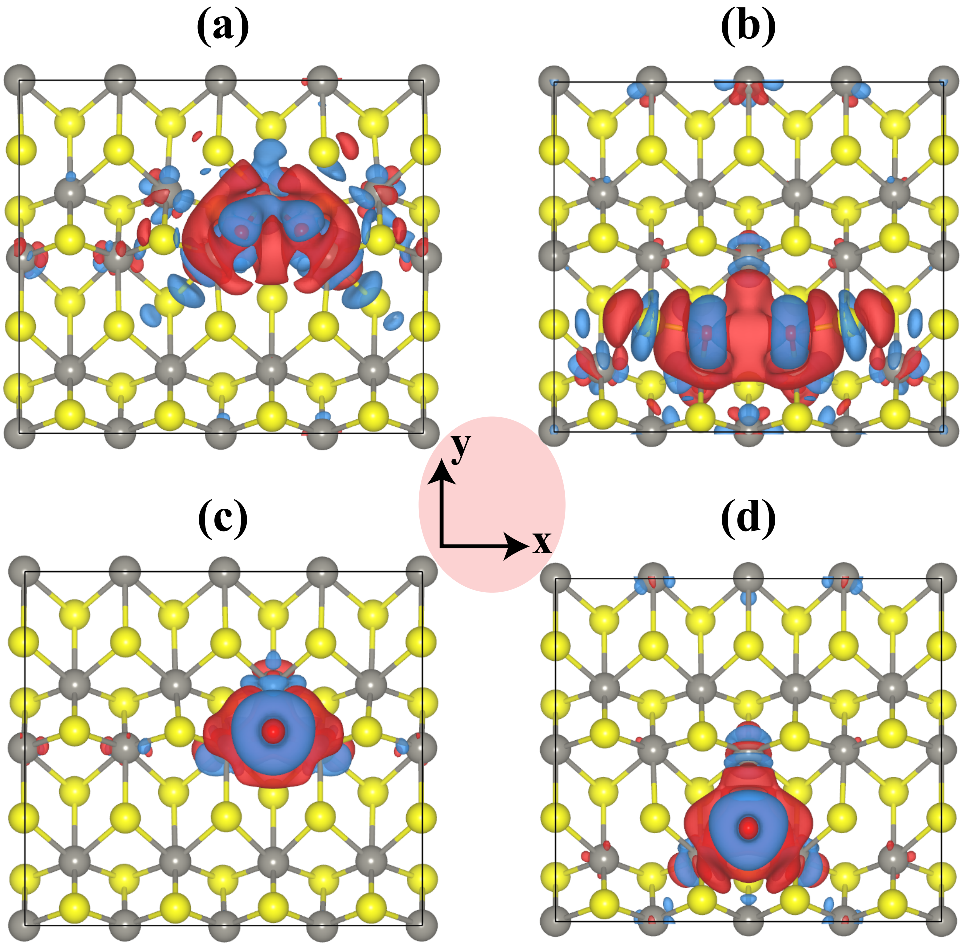

Figure 12. Isosurfaces of electronic charge density difference, $\Delta\rho(\mathbf{r})$, for **(a)** Te(1)-O_2, **(b)** Te(2)-O_2 substitutions with parallel orientation, **(c)** Te(1)-O_2, **(d)** Te(2)-O_2 substitutions with vertical orientation. Excess electronic charge is indicated in blue whereas deprivation one is in red. The isosurfaces are set to 0.0015 e/Å^3.

Section S7: Charge transfer analysis in the defects other than substitution

Here, we present the isosurfaces of interatomic electronic charge density difference ($\Delta\rho(\mathbf{r})$) between W and Te atoms. This was obtained by subtracting the charge densities residing at the atomic sites from the total electronic charge density of the system ($\rho_T(\mathbf{r})$). (Fig. 13). Since the electronegativity of Te (2.1) is higher that that of W (1.7), electrons are transferred from W to Te in equilibrium. By the effects of the defects considered, these charges are redistributed among neighboring atoms as seen in Fig. 13.

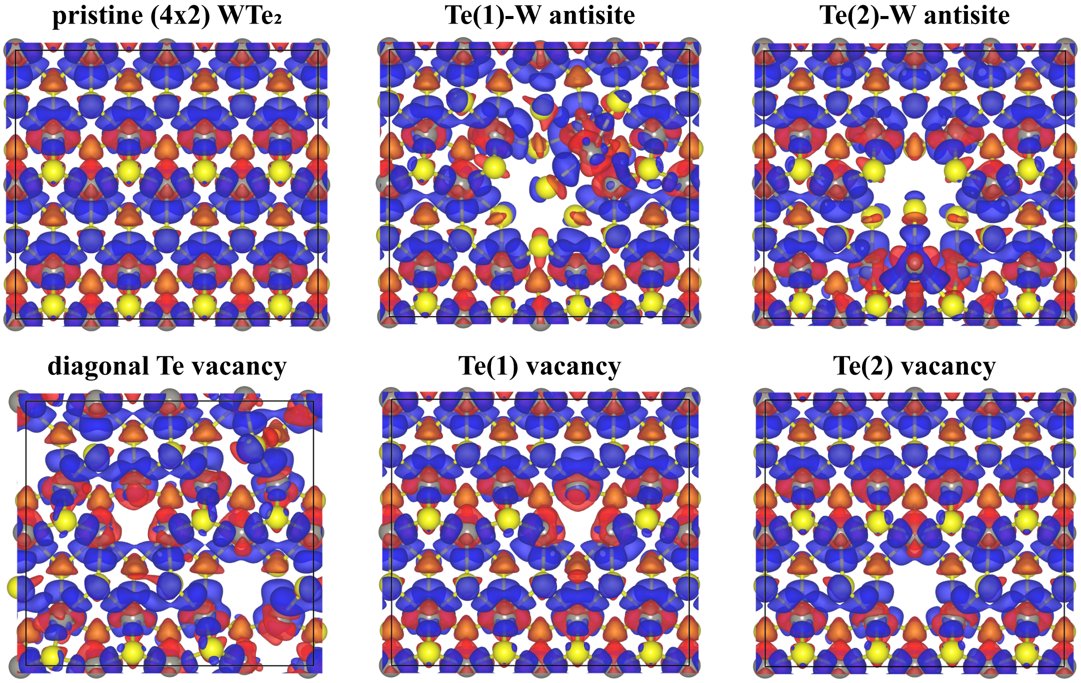

Figure 13. Isosurfaces of electronic charge density difference, $\Delta\rho(\mathbf{r})$, for the defects other than substitution. Excess electronic charge is indicated in blue whereas deprivation one is in red. The isosurfaces are set to 0.0055 e/Å^3.

Section S8: Electronic density of states plots of defective 1L 1T' WTe_2

Here, we present the total (TDOS) and atomic-orbital projected (PDOS) electronic density of states plots of defective 1L 1T' WTe_2 structures calculated using PBE functional including SOC, electronic energy band structures of which are presented in main part of the study in Figure 7.

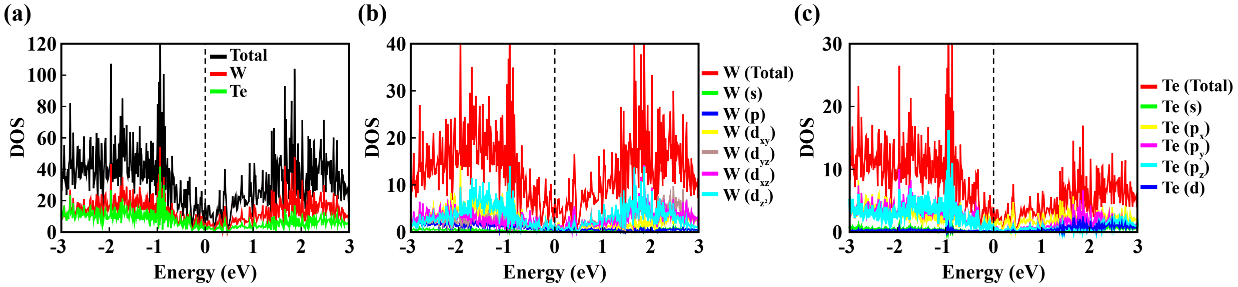

Figure 14. Total (TDOS) and atomic-orbital projected (PDOS) electronic density of states of 1L 1T' WTe_2 with diagonal Te vacancy defect. Zero of energy is set at the Fermi level shown by black-dashed line.

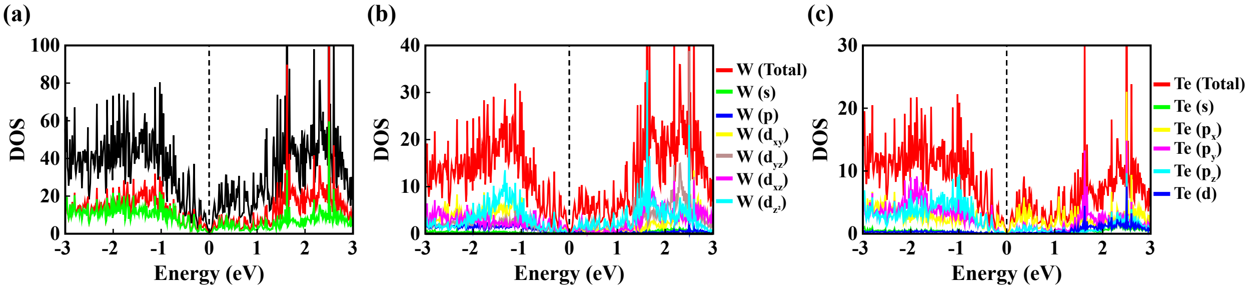

Figure 15. Total (TDOS) and atomic-orbital projected (PDOS) electronic density of states of 1L 1T' WTe_2 with Te(1) vacancy defect. Zero of energy is set at the Fermi level shown by black-dashed line.

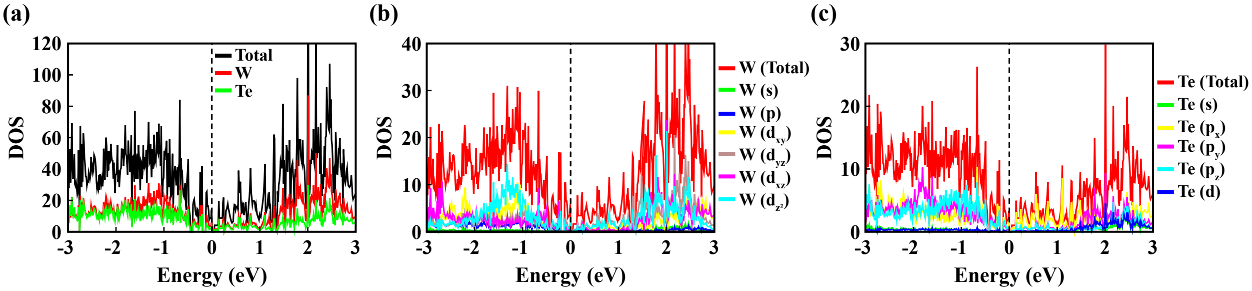

Figure 16. Total (TDOS) and atomic-orbital projected (PDOS) electronic density of states of 1L 1T' WTe_2 with Te(2) vacancy defect. Zero of energy is set at the Fermi level shown by black-dashed line.

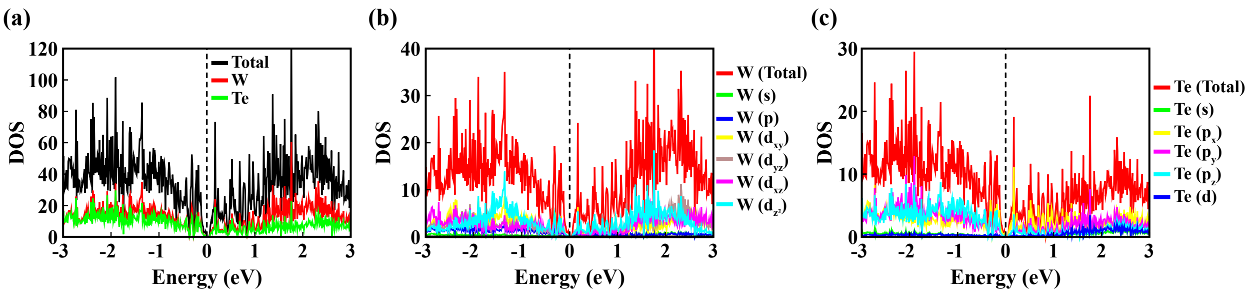

Figure 17. Total (TDOS) and atomic-orbital projected (PDOS) electronic density of states of 1L 1T' WTe_2 with Te(1)-W antisite defect. Zero of energy is set at the Fermi level shown by black-dashed line.

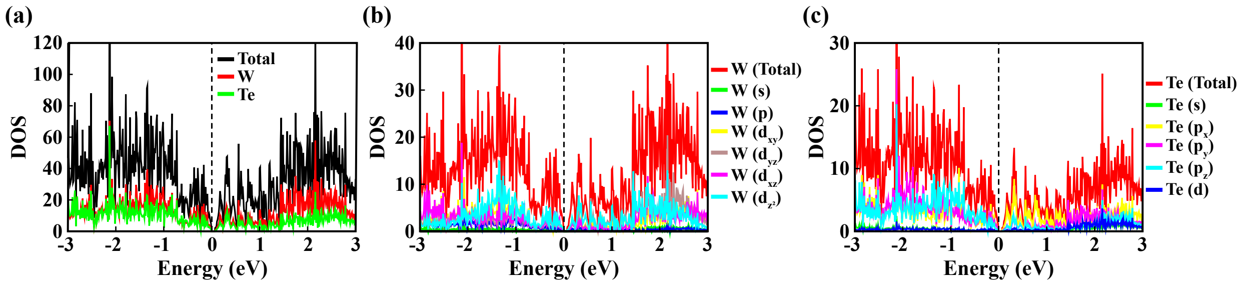

Figure 18. Total (TDOS) and atomic-orbital projected (PDOS) electronic density of states of 1L 1T' WTe_2 with Te(2)-W antisite defect. Zero of energy is set at the Fermi level shown by black-dashed line.

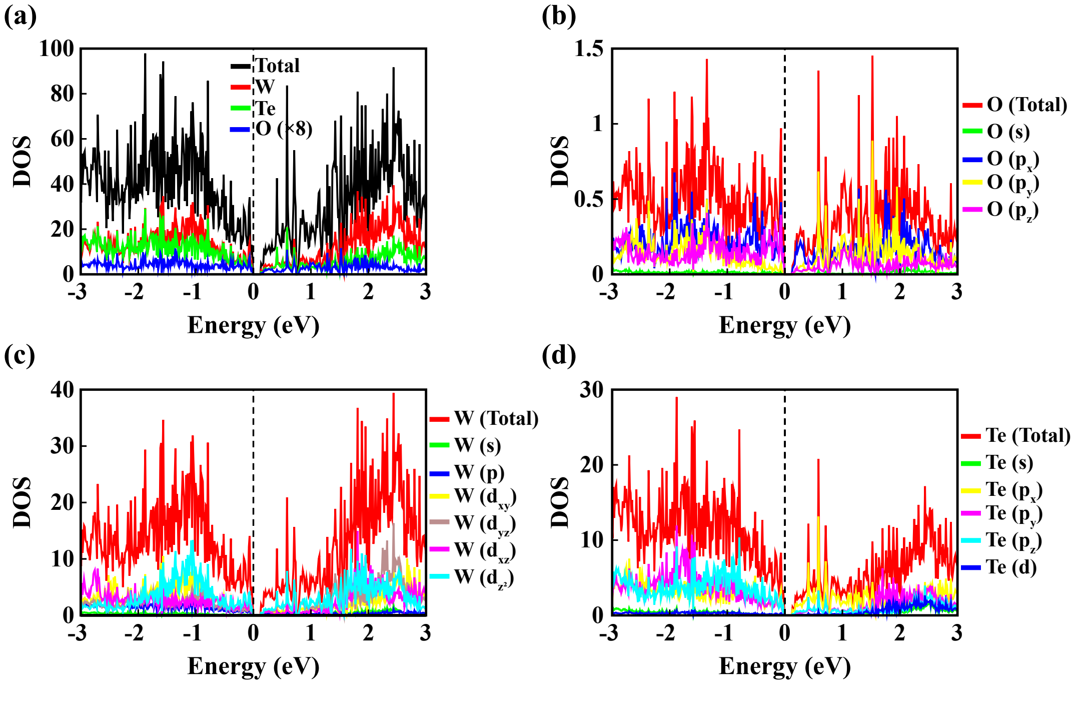

Figure 19. Total (TDOS) and atomic-orbital projected (PDOS) electronic density of states of 1L 1T' WTe_2 with Te(1)-O_2 substitution (parallel to surface orientation) defect. Zero of energy is set at the Fermi level shown by black-dashed line.

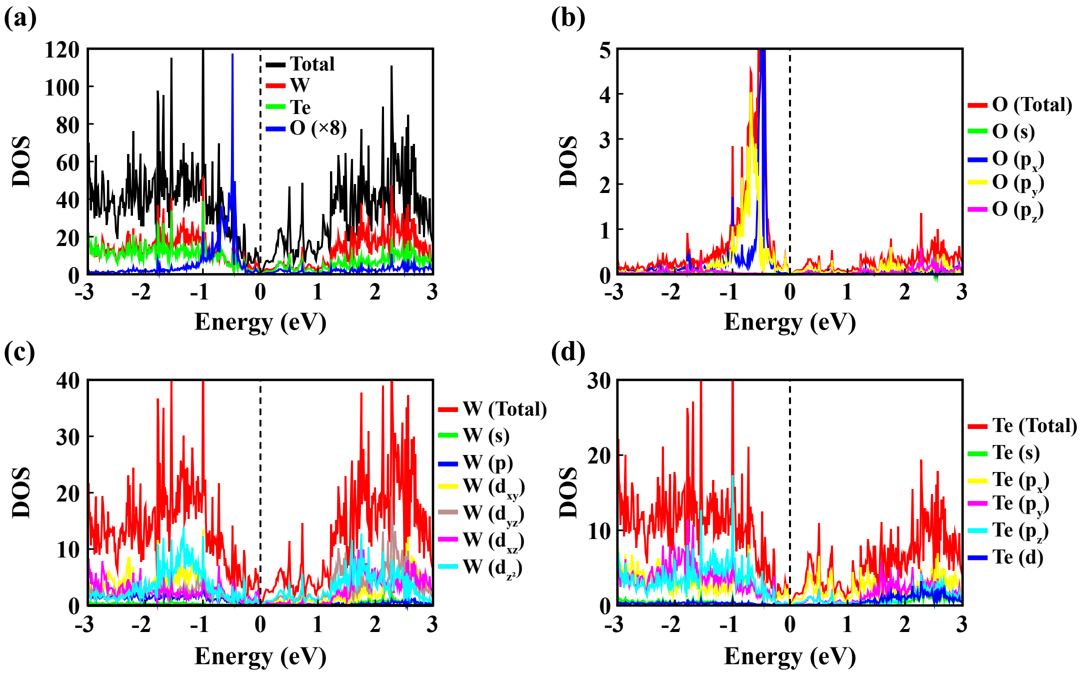

Figure 20. Total (TDOS) and atomic-orbital projected (PDOS) electronic density of states of 1L 1T' WTe_2 with Te(1)-O_2 substitution (vertical to surface orientation) defect. Zero of energy is set at the Fermi level shown by black-dashed line.

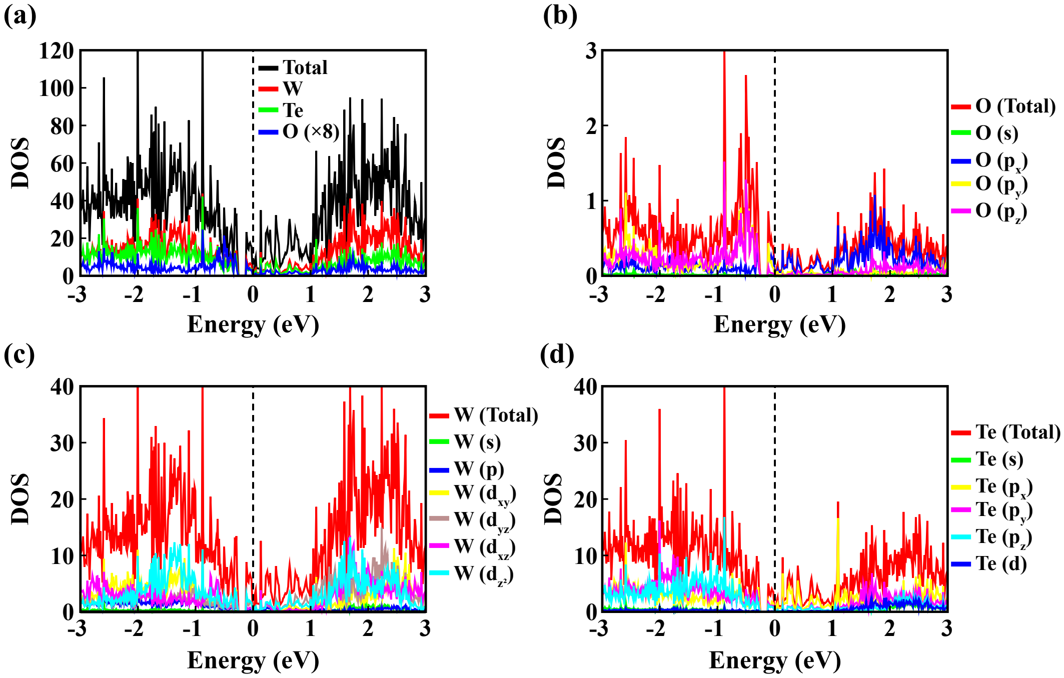

Figure 21. Total (TDOS) and atomic-orbital projected (PDOS) electronic density of states of 1L 1T' WTe_2 with Te(2)-O_2 substitution (parallel to surface orientation) defect. Zero of energy is set at the Fermi level shown by black-dashed line.

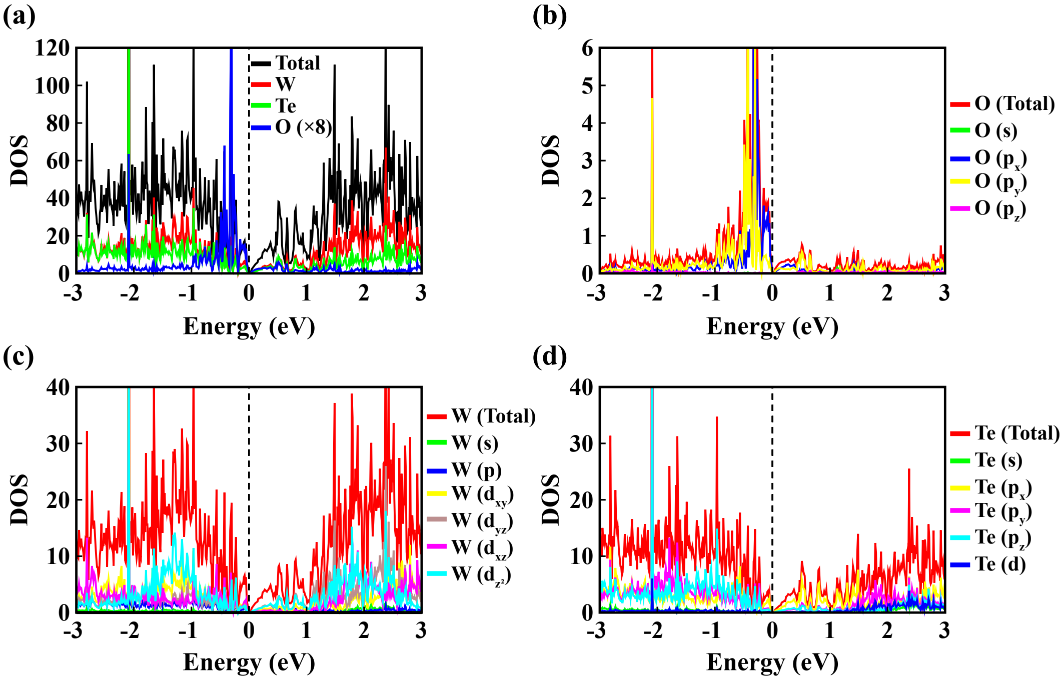

Figure 22. Total (TDOS) and atomic-orbital projected (PDOS) electronic density of states of 1L 1T' WTe_2 with Te(2)-O_2 substitution (vertical to surface orientation) defect. Zero of energy is set at the Fermi level shown by black-dashed line.

Section S9: Thermoelectric properties of defective 1L 1T' WTe_2

Here, we present the Seebeck coefficient (S) and relaxation time dependent electrical conductivity (σ/τ_0) as a function of chemical potential calculated at various temperatures for diagonal Te vacancy, Te(1)-W antisite, Te(2)-W antisite, Te(1)-O_2 substitution with vertical orientation and Te(2)-O_2 substitution with vertical orientation defects (Figs. 23 and 24). Besides, the Seebeck coefficient and relaxation time dependent electrical conductivity for all types of defects considered are plotted as a function of temperature within the range of 100-400 K for various chemical potentials in Figs. 25 and 26.

It is known that the thermoelectric performance of materials is commonly specified by a dimensionless quantity, so-called figure-of-merit (ZT), described as $ZT = \sigma S^2 T/\kappa$. Here κ is thermal conductivity, which is the sum of the electrical and lattice thermal conductivity, and T is the absolute temperature. In order to obtain the optimum material for thermoelectric applications, high ZT is required. Satisfying this necessity can be handled with low κ or high $S^2\sigma$ values. Here, $S^2\sigma$ is generally described as power factor (PF). In addition to the Seebeck coefficient and electrical conductivity, we present the relaxation time dependent power factor (PF=$S^2\sigma/\tau$) plots of defective 1L 1T' WTe_2 in Fig. 27. Looking at the figure of power factor, it turns out that the largest thermoelectric power factor at 400 K is reached for pristine WTe_2 and the PF is lower for all defective structures. This suggests that defect engineering has negative impact on the thermoelectric power factor, and that in order to meet the high ZT requirement low κ is vital for defective WTe_2 structures.

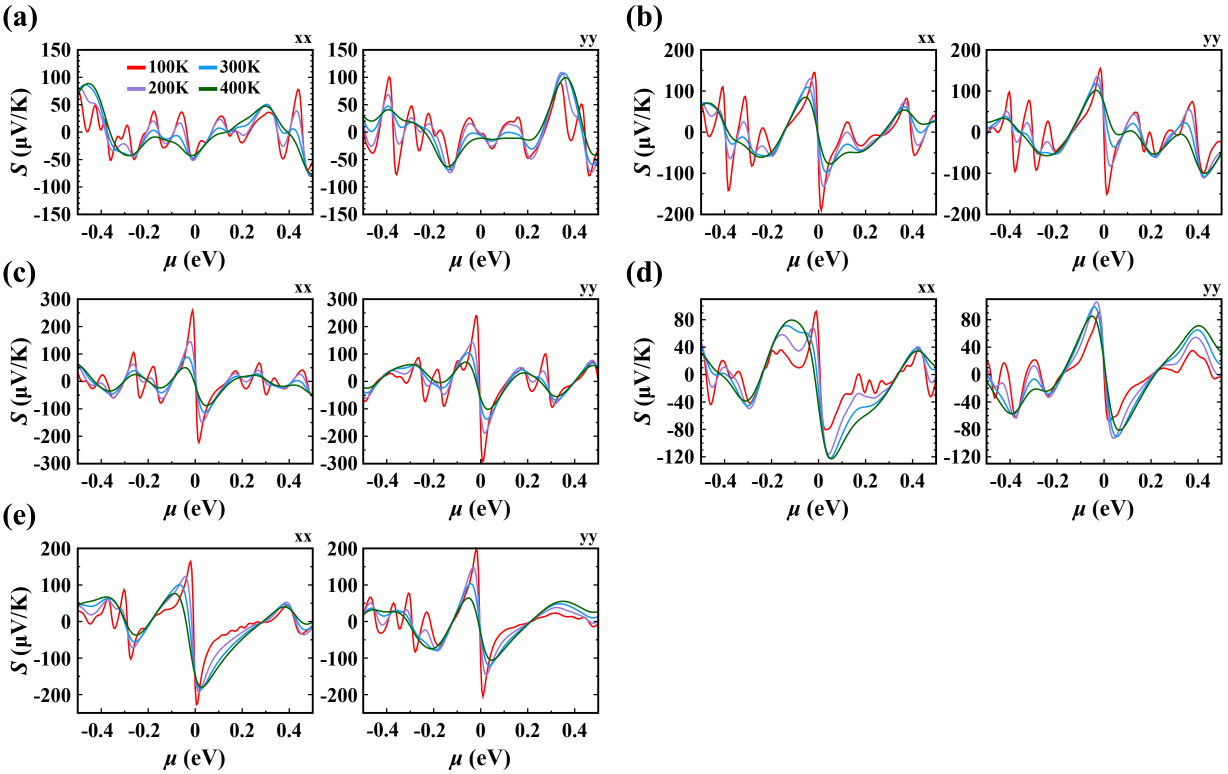

Figure 23. Seebeck coefficient (S) calculated as a function of chemical potential (μ) at various temperatures along xx and yy lattice directions for **(a)** diagonal Te vacancy, **(b)** Te(1)-W antisite, **(c)** Te(2)-W antisite, **(d)** Te(1)-O_2 substitution with vertical orientation and **(e)** Te(2)-O_2 substitution with vertical orientation.

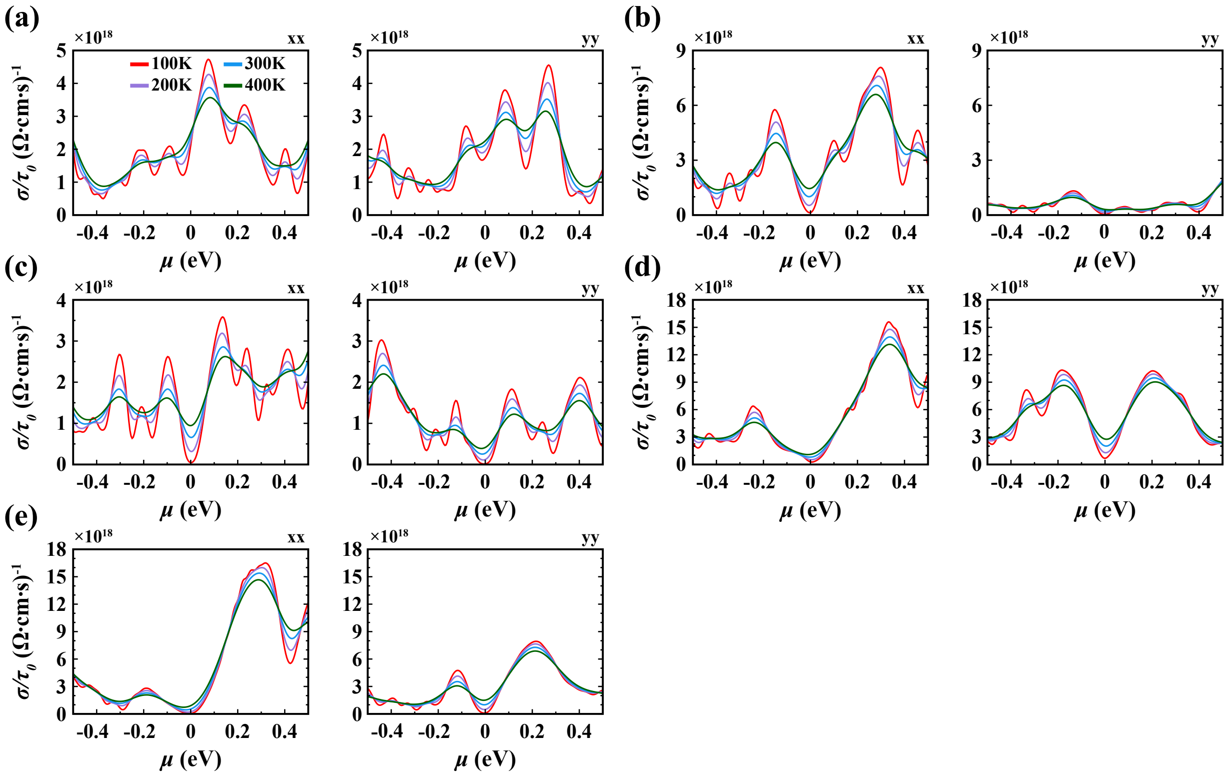

Figure 24. Electrical conductivity with respect to relaxation time (σ/τ_0) calculated as a function of chemical potential (μ) at various temperatures along *xx* and *yy* lattice directions for **(a)** diagonal Te vacancy, **(b)** Te(1)-W antisite, **(c)** Te(2)-W antisite, **(d)** Te(1)-O_2 substitution with vertical orientation and **(e)** Te(2)-O_2 substitution with vertical orientation.

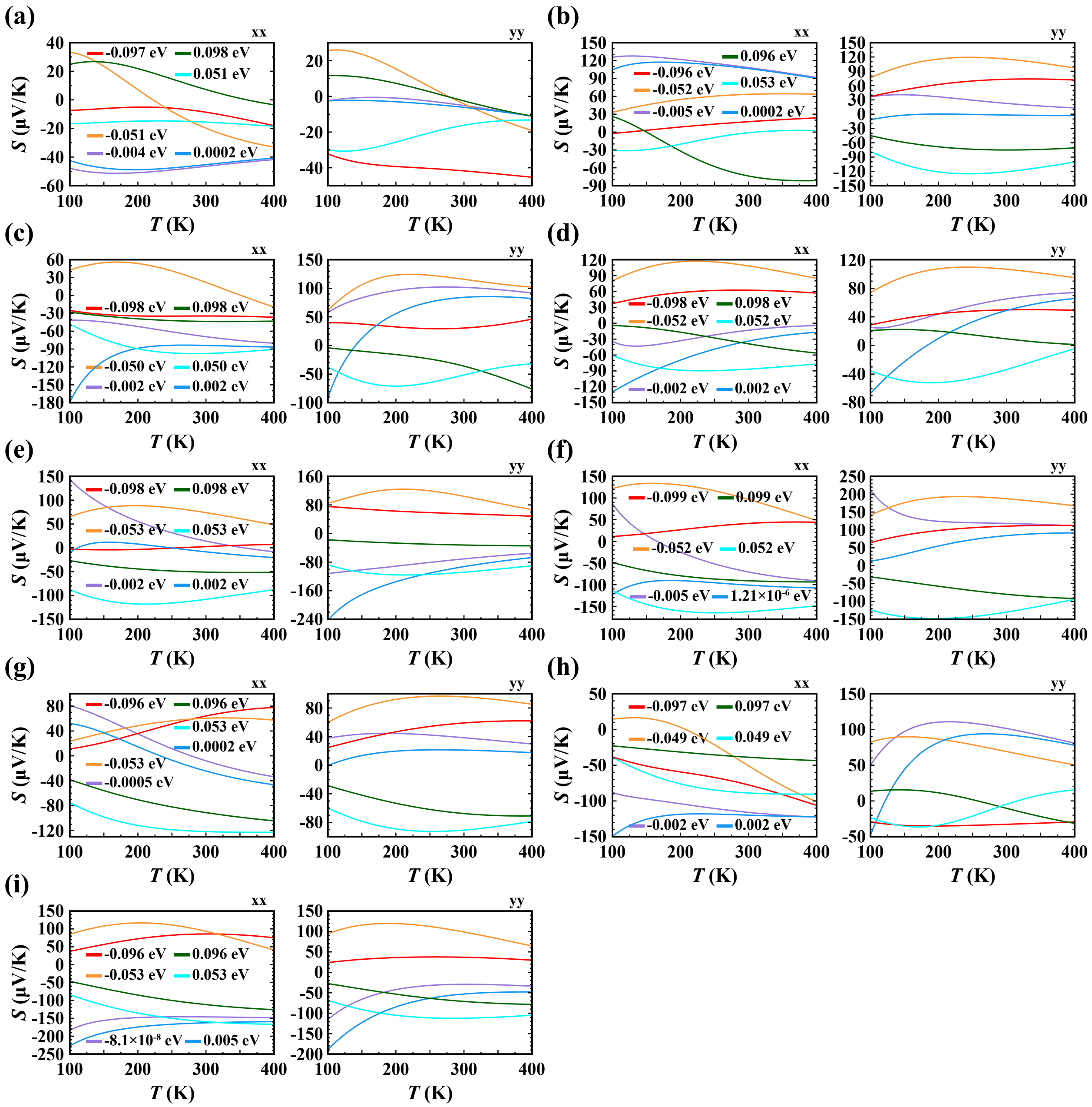

Figure 25. Seebeck coefficient (S) calculated as a function of temperature (T) within the range of 100-400 K at various chemical potentials for defective 1L 1T' WTe_2 along *xx* and *yy* lattice directions.

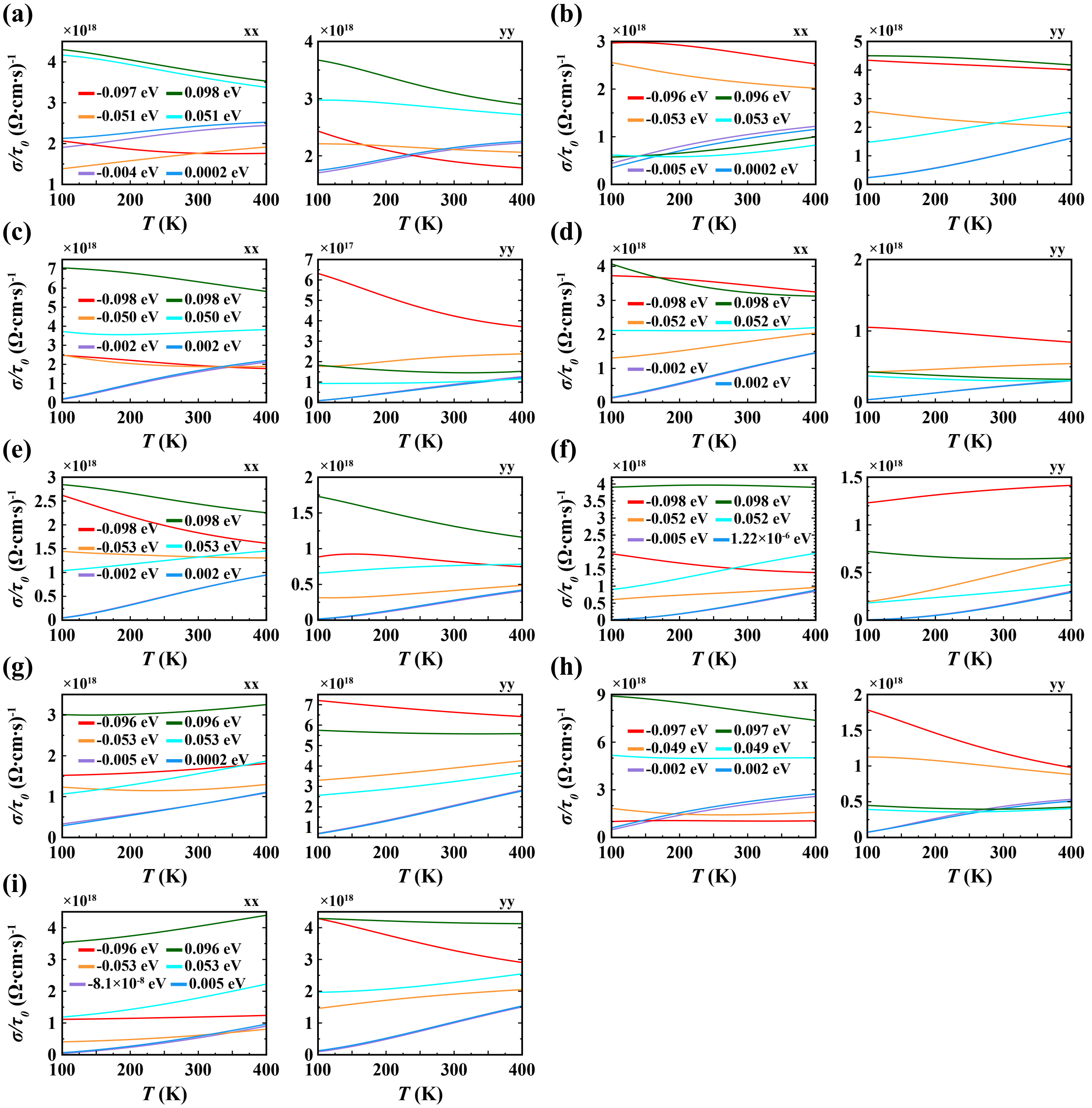

Figure 26. Electrical conductivity with respect to relaxation time (σ/τ_0) calculated as a function of temperature (T) within the range of 100-400 K at various chemical potentials for defective 1L 1T' WTe_2 along *xx* and *yy* lattice directions.

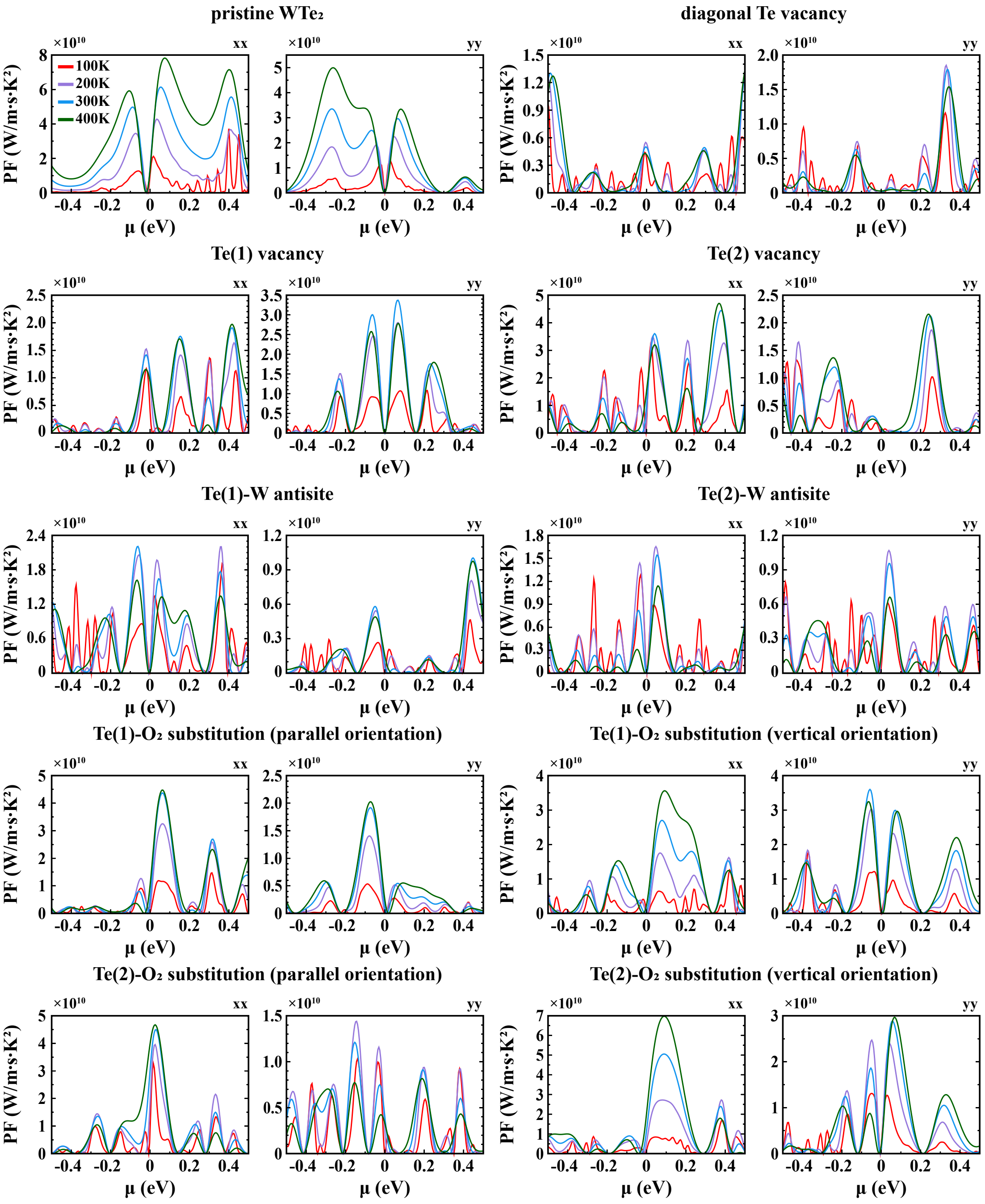

Figure 27. Relaxation time dependent thermoelectric power factor (PF=$S^2\sigma/\tau$) for defective 1L 1T' WTe_2.

References

1. Henkelman, G., Arnaldsson, A. & Jónsson, H. A fast and robust algorithm for bader decomposition of charge density. *Comput. Mater. Sci.* **36**, 354–360, DOI: 10.1016/j.commatsci.2005.04.010 (2006).
